# From inflammatory activation to fibrotic remodeling: the central role of macrophage heterogeneity in frozen shoulder

**DOI:** 10.3389/fimmu.2026.1732435

**Published:** 2026-02-04

**Authors:** Hao Liang, Jianmin Liu

**Affiliations:** 1 College of Acupuncture-Moxibustion and Orthopedics, Hubei University of Chinese Medicine, Wuhan, Hubei, China; 2 Hubei Shizhen Laboratory, Hubei University of Chinese Medicine, Wuhan, Hubei, China

**Keywords:** fibrosis, frozen shoulder, immune microenvironment, inflammation, macrophage polarization, macrophages

## Abstract

Frozen shoulder (FS) is a condition primarily marked by chronic inflammation and progressive fibrosis of the glenohumeral capsule. Clinically, it presents with persistent shoulder pain and limited joint mobility, often leading to impaired upper limb function and reduced quality of life. In recent years, research on the molecular mechanisms of fibrosis in FS has deepened; however, there remains a lack of systematic focus on the dynamic regulation of the immune microenvironment, particularly the role of macrophage heterogeneity. The remarkable functional plasticity of macrophages allows them to play dual roles in inflammatory responses and tissue repair, with the sequential transformation of their phenotypes and functions potentially governing various stages of FS development. This review focuses on the dynamic evolution of macrophage function and polarization states during FS progression. This review systematically outlines the key roles of these cells in inflammatory responses, fibrosis progression, and their signaling interactions with fibroblasts. We systematically assessed the critical signaling networks that regulate macrophage recruitment and differentiation, emphasizing the core roles of the C-C motif chemokine ligand 2 (CCL2)/C-C chemokine receptor type 2 (CCR2) chemotactic axis and tissue mechanical signals in this process. Additionally, we explored how the synergy between immune signaling and mechanotransduction maintains the pathogenic activation state of macrophages, potentially forming a positive feedback loop for fibrosis. Targeting these upstream inputs holds promise as a potential intervention to disrupt the link between inflammation and fibrosis. A deeper understanding of the regulatory mechanisms governing macrophage heterogeneity will lay the foundation for developing more targeted therapeutic approaches for FS in the future.

## 1 Introduction

Frozen shoulder (FS), also referred to as adhesive capsulitis of the shoulder, is a debilitating condition characterized by shoulder pain and a progressive limitation of both active and passive movement in the glenohumeral joint (1). Epidemiological studies suggest that FS affects approximately 2%–5% of the global population, with its prevalence increasing (1, 2). This condition predominantly manifests in individuals aged 40–60 years, with a peak incidence around age 50, and is approximately 1.23 times more prevalent in women than in men (3, 4). Recent Mendelian randomization evidence further suggests that epigenetic age acceleration is bi-directionally associated with age-related musculoskeletal disorders, supporting a more active role of biological aging in chronic degeneration (5). In line with this age-related biological context, although FS has traditionally been considered a self-limiting disease, clinical studies have demonstrated that a substantial number of patients experience long-term functional impairment even after symptom improvement (1, 6, 7). Furthermore, persistent pain and restricted movement frequently lead to adverse emotional responses, such as anxiety, fear, and depression, which can affect treatment adherence and rehabilitation outcomes to some extent (8). Overall, FS not only severely diminishes the quality of life of patients but also significantly increases the medical and economic burdens on both individuals and society.

**Figure 1:**
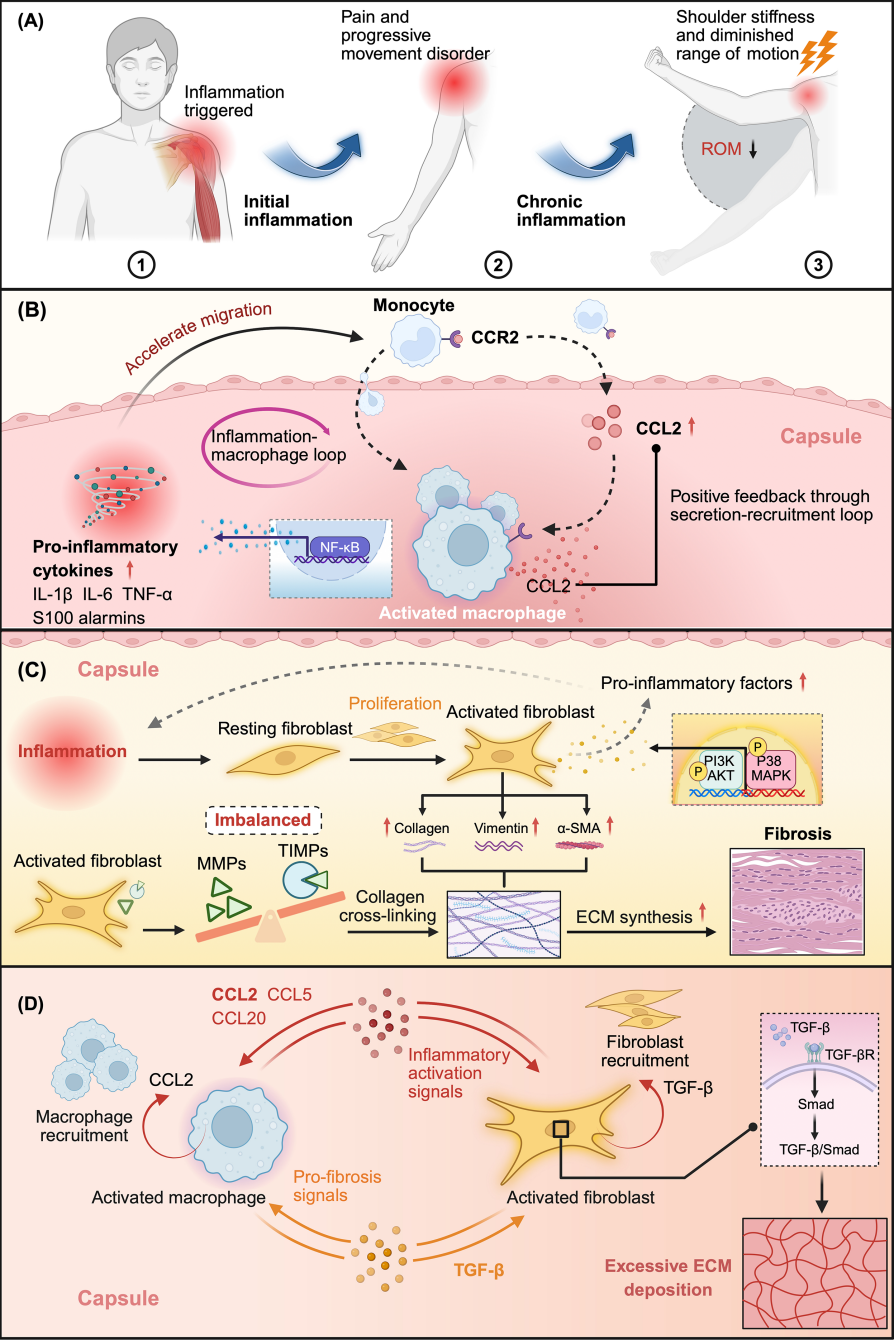
Macrophage activation driven by inflammation orchestrates fibrotic remodeling in FS. **(A)** FS initiation and progression. Local irritation or micro-injury triggers inflammation within the glenohumeral capsule, resulting in pain and initiating chronic inflammatory processes that culminate in capsular thickening, stiffness, and restricted movement. **(B)** Macrophage recruitment and amplification. Elevated levels of pro-inflammatory cytokines and alarmins in the inflamed capsule recruit circulating monocytes, which differentiate into macrophages at the lesion site. In response to inflammatory cues, stromal cells upregulate CCL2 expression, forming a chemotactic gradient that drives the influx of CCR2^+^ macrophages. Activated macrophages further release cytokines and CCL2 through NF-κB signaling, reinforcing the CCL2/CCR2 axis-centered regulatory network, which strengthens immune activation and maintains an inflammatory microenvironment. **(C)** Fibroblast activation and matrix remodeling. Persistent inflammatory stimuli lead to fibroblast proliferation and activation, characterized by elevated levels of α-SMA, vimentin, and collagen production. The PI3K/AKT and p38 MAPK signaling pathways enhance the inflammatory capabilities of fibroblasts and sustain the release of pro-fibrotic mediators. Additionally, an imbalance between MMPs and TIMPs impedes ECM degradation, promoting collagen cross-linking and capsular fibrosis progression. **(D)** Macrophage–fibroblast interplay in the continuous spectrum from inflammation to fibrosis. Macrophage-derived pro-inflammatory factors activate fibroblasts, which in turn produce chemokines that facilitate macrophage accumulation at the lesions. Macrophages maintain their presence via autocrine CCL2, while macrophage-derived TGF-β activates the TGF-β/Smad pathway in fibroblasts, promoting ECM deposition. Fibroblast-derived TGF-β further reinforces fibroblast activation and enhances profibrotic macrophage activity, creating reciprocal loops that amplify the continuous spectrum of inflammation to fibrosis in FS. *Created in BioRender. Liang*, (H) *(2025)*https://BioRender.com/qh6mebt. FS, frozen shoulder; IL-1β, interleukin-1 beta; IL-6, interleukin-6; TNF-α, tumor necrosis factor alpha; CCL2, C-C motif chemokine ligand 2; CCR2, C-C motif chemokine receptor 2; NF-κB, nuclear factor kappa-light-chain-enhancer of activated B cells; α-SMA, alpha-smooth muscle actin; PI3K, phosphatidylinositol 3-kinase; AKT, protein kinase B; MAPK, mitogen-activated protein kinase; MMP, matrix metalloproteinase; TIMP, tissue inhibitor of metalloproteinase; ECM, extracellular matrix; TGF-β, transforming growth factor beta; Smad, mothers against decapentaplegic homolog.

Current evidence indicates that FS is a chronic musculoskeletal disorder predominantly characterized by capsular contracture, with dynamic progression in which inflammation initiates and fibrosis consolidates the pathology (9–11). Clinical and histopathological studies have generally classified the disease course into three stages: early inflammatory pain hypersensitivity, intermediate frozen, and late thawing, each exhibiting distinct pathological features and cellular activities (12, 13) (**Figure 1A**). Although the precise etiology remains unclear, accumulating evidence indicates that an early inflammatory response, potentially triggered by repetitive strain, trauma, rotator cuff injury, or subacromial impingement, activates local immunity and induces the release of inflammatory and growth factors, thereby promoting fibroblast activation, disrupting extracellular matrix (ECM) homeostasis, and accelerating fibrosis (6, 14, 15).

In the context of this pathological progression, macrophages serve as crucial mediators connecting inflammation with fibrotic alterations. Their context-dependent plasticity enables them to undergo phenotypic and functional shifts across various disease stages, thereby influencing downstream outcomes in FS (16–18). Within the classical paradigm, resting macrophages (M0) can polarize into either pro-inflammatory M1-like (classical activation) or pro-restorative M2-like (alternative activation) macrophages. These polarized macrophages actively modulate the local immune–stromal microenvironment by secreting a range of pro-inflammatory and pro-fibrotic mediators, thus linking the initial inflammatory response to fibrotic progression (19–21). Consequently, unresolved inflammation promotes fibroblast activation and excessive ECM deposition (22–24). Single-cell analyses have identified specialized macrophage subsets, including MER tyrosine kinase (MERTK)-positive clusters, which exhibit transcriptional programs associated with the negative regulation of inflammation and may facilitate disease resolution (18). This highlights the pivotal role of macrophage plasticity in determining the inflammatory and fibrotic trajectories of FS. In addition, upstream signals that regulate macrophage homing and programming, such as the C-C motif chemokine ligand 2 (CCL2)/C-C motif chemokine receptor 2 (CCR2) axis and persistent mechanical cues, including matrix stiffness and tissue tension, are likely to affect the rate and extent of the transition from inflammation to fibrosis (18, 25). Despite their importance, these upstream regulatory mechanisms remain insufficiently characterized, warranting an integrative synthesis.

While current research indicates that macrophages may play a crucial regulatory role in FS fibrosis progression, there remains a significant lack of mechanistic studies exploring phenotypic heterogeneity and functional states during inflammation, fibroblast activation, and ECM accumulation. This gap is evident in the understanding of the upstream regulatory pathways and effects of the mechanical microenvironment. This review examines the functional evolution of macrophages during FS, highlighting their roles in the inflammatory and fibrotic stages. This review emphasized the influence of CCL2/CCR2 chemotactic signaling and mechanical stimulation on macrophage polarization and functional expression, and explored the potential applications of related intervention strategies.

## 2 Macrophage regulation of the inflammation–fibrosis continuum in FS

### 2.1 Macrophage orchestration of early inflammation in FS

The initiation of FS is distinguished by chronic inflammation within the glenohumeral capsule, a process widely acknowledged as being essential for the advancement of fibrosis. Initial research has revealed that the expression of various pro-inflammatory factors, such as interleukin (IL)-1α, IL-1β, IL-6, tumor necrosis factor (TNF)-α, and inducible cyclooxygenase (COX)-1 and COX-2, is typically increased in capsule tissue. The activation of COX-2 notably leads to an increased production of prostaglandin E2 (PGE2), which exacerbates the inflammatory cascade, aggravates tissue damage, and fosters a pro-inflammatory environment conducive to fibrotic outcomes (26, 27). Numerous studies have shown that FS-related inflammation generally presents a low-intensity but persistent expression pattern. This persistent inflammation not only impedes the healing of micro-injuries in the capsule but also continuously provides signals that trigger the onset of subsequent fibrosis (28, 29) (**Figure 1B**).

In this context, macrophages emerge as pivotal immune cells during the early stages of inflammatory responses. Hand et al. (16) highlighted in their research that chronic inflammation, marked by significant macrophage infiltration, is frequently observed in the capsules of FS patients. This suggests that ongoing inflammation is a crucial factor in the pathological progression of FS and may be influenced by immune mechanisms. Recent advancements in single-cell RNA sequencing have deepened the understanding of macrophages in FS, confirming their status as the most prevalent immune-infiltrating cells in lesions and underscoring their potential central role in exacerbating inflammation and driving pathological progression (17, 30, 31). Akbar et al. (15) also observed that CD64^+^ macrophages are the dominant infiltrating population in the early stages of FS, with their activation and aggregation possibly forming a critical foundation for the persistence of inflammation. In summary, macrophages are highly concentrated in FS inflammatory lesions, and their activation may be a pivotal link to sustained amplification of inflammation.

The substantial accumulation of macrophages within the lesion area is not an isolated phenomenon but is precipitated by persistent stimulation of the local inflammatory milieu. Previous research has demonstrated that cytokines present in the local inflammatory microenvironment can induce peripheral monocyte migration into the glenohumeral capsule and differentiate into inflammatory macrophages. These populations subsequently release large amounts of pro-inflammatory mediators, including IL-1β, IL-6, TNF-α, and alarmins such as S100A8/A9, thereby intensifying and sustaining inflammatory activity (32, 33) It is crucial to underscore that the continuous recruitment and activation of macrophages serve as the primary driving force for amplifying inflammation. In addition to the direct chemotactic effects of broad-spectrum inflammatory mediators, macrophage-directed migration pathways, exemplified by the CCL2/CCR2 axis, provide a fundamental molecular basis for the sustained supply of macrophages and maintenance of inflammation. CCL2 can be secreted by macrophages, fibroblasts, and synovial cells under inflammatory or injury conditions. Its principal receptor, CCR2, is a seven-transmembrane G protein-coupled receptor predominantly expressed on the surfaces of monocytes and macrophages. The interaction between the two activates downstream signaling pathways, facilitating the directional migration and tissue infiltration of monocytes/macrophages, and sustaining the recruitment and activation of immune cells (15, 34–36). In the lesions, CCL2 overexpression enhances macrophage recruitment, thereby enabling persistent inflammation. An increase in macrophages is also accompanied by the upregulation of chemokines, such as CCL2 and CCL20, further attracting immune cells and establishing a positive feedback loop for inflammation (15) (**Figure 1B**).

In addition to classic cytokines, the abnormal expression of various alarmins may also contribute to the pathological progression of FS by activating macrophages. Molecules such as high mobility group box protein B1 (HMGB1), S100A8/A9, and IL-33, which are highly expressed in the capsule tissue of FS, exhibit significant spatial overlap between their infiltration areas and collagen deposition sites. This suggests a close structural association between inflammation and fibrosis (33). Among these, HMGB1 binds to the receptor for advanced glycation end products (RAGE), leading to activation of the nuclear factor kappa-B (NF-κB) pathway and subsequent upregulation of inflammatory factors, which promote macrophage activation and sustain inflammation (37–40) (**Figure 1B**).

In summary, macrophages are pivotal orchestrators in the initiation, amplification, and persistence of early inflammation in FS, and their sustained activation and recruitment represent a critical link between inflammatory responses and fibrotic remodeling.

### 2.2 Fibroblast effector functions underlying fibrosis in FS

Fibroblasts, as core units connecting tissue structure and function, play crucial roles in the spatial distribution and activation states during the progression of FS fibrosis. The glenohumeral capsule is primarily composed of an inner synovial membrane and an outer layer of dense connective tissue. Notably, the synovium is the initial region affected by the disease, undergoing significant cellular and matrix alterations during the early stages of FS onset (41, 42). Synovial fibroblasts, also referred to as fibroblast-like synoviocytes, are the predominant cell type within the synovium and possess both matrix synthesis and immune regulatory functions (25). Immunohistochemical analysis by Akbar et al. (10) revealed that activation markers of fibroblasts, such as CD248, CD146, vascular cell adhesion molecule-1 (VCAM), and podoplanin (PDPN), were significantly upregulated in the lesion areas of patients with FS, indicating a pathologically hyperactive state. Further *in vitro* investigations have demonstrated that primary fibroblasts isolated from the capsules of patients with FS exhibit enhanced proliferative capacity, differentiation tendency, and collagen synthesis activity, supporting the hypothesis that fibroblast expansion and sustained activation are critical features of early FS fibrosis (43, 44). Collectively, these findings highlight the effector role of fibroblasts in FS at both tissue and cellular levels.

In the inflammatory microenvironment of FS, persistent activation of fibroblasts relies heavily on the continuous influx of inflammatory signals. Previous studies have confirmed that pro-inflammatory mediators, such as IL-1β, TNF-α, and IL-6, are significantly upregulated in the capsule tissue and may serve as key initiating signals for the transformation of the fibroblast phenotype (28, 45, 46). Notably, IL-1β is not only elevated at the tissue level, but has also been shown *in vitro* to directly induce fibrosis-promoting effects in fibroblasts from patients with FS or healthy donors. These effects include enhanced proliferation and migration abilities and upregulation of fibrosis markers such as collagen type I (COL1), vimentin, and α-smooth muscle actin (α-SMA) (10). Further mechanistic research has revealed that IL-1β can promote the sustained expression of pro-fibrotic genes and enhance the fibroblast stress response and collagen synthesis ability by activating the p38 mitogen-activated protein kinase (p38 MAPK) pathway (45). Additionally, TNF-α primarily contributes to the early amplification of the synovial inflammatory response in FS, whereas IL-6 can enhance the activation level and collagen synthesis activity of synovial fibroblasts by activating the phosphatidylinositol 3-kinase (PI3K) - protein kinase B (AKT) signaling pathway, thereby promoting the conversion of inflammatory signals into a fibrotic response (28, 46) (**Figure 1C**). These studies reveal a common pathway through which multiple inflammatory factors synergistically induce fibroblast pro-fibrotic programs, forming a crucial link between early inflammation and the fibrosis transition of FS. Whether fibroblasts possess the ability to maintain and amplify their activated state after receiving inflammatory signals has become a key issue in understanding the persistence of their fibrotic effects.

Extensive research evidence indicates that inflammation serves as the initial activation signal for fibroblasts, which can utilize endogenous regulatory mechanisms to sustain and amplify their activation state, thereby establishing a stable and persistent pro-fibrotic response. Research in organizational studies has shown that in the lesion area of the capsule, approximately half of the cells express typical alarm protein molecules, such as HMGB1 and IL-33, which are mainly found in fibroblasts and endothelial cells. These observations imply an acquired pro-inflammatory phenotype of structural cells under chronic inflammatory conditions (33). Further investigations have revealed that activated fibroblasts can detect external inflammatory stimuli and continuously release various inflammatory products through autocrine mechanisms, including IL-6, IL-8, CCL20, and reactive oxygen species (ROS). This process enhances the sensitivity of surrounding tissues to inflammatory stimuli while maintaining the fibroblast activation state (10, 45) (**Figure 1C**). This cellular endogenous feedback establishes a stable activation circuit, which can sustain a pro-fibrotic phenotype in the absence of continuous external stimuli and may provide a persistent signaling source for the progression from inflammation to fibrosis in FS.

Fibroblasts serve as pivotal agents in ECM remodeling during FS progression, directing both matrix synthesis and degradation. Within these regulatory pathways, matrix metalloproteinases (MMPs) and tissue inhibitors of metalloproteinases (TIMPs) function as crucial mediators that establish the equilibrium of ECM turnover (47, 48) (**Figure 1C**). In the capsules affected by FS, the upregulation of MMP-2, MMP-9, and MMP-12 indicates an activated degradative response (29, 49, 50), whereas the aberrant elevation of TIMP-1 inhibits MMP activity, diminishes matrix clearance, and promotes persistent ECM accumulation (11, 27, 51). Evidence from synovial fibrosis in osteoarthritis (OA) similarly suggests that dysregulation of the MMP/TIMP axis can perpetuate fibrotic progression, with fibroblasts positioned centrally within this regulatory network (52).

### 2.3 Macrophage–fibroblast interactions underpin fibrosis progression in FS

Chronic inflammation is instrumental in the activation of fibroblasts, highlighting their responsiveness to the development of FS fibrosis. In this context, macrophages as the predominant immune cell type, not only orchestrate the establishment of the inflammatory microenvironment, but also facilitate the persistent amplification of the fibrotic response through bidirectional signaling with fibroblasts.

The Medzhitov team (53) pioneered the dual cell loop theory in 2018, which suggests that macrophages and fibroblasts can establish stable intercellular interactions through growth factor exchange, providing a conceptual basis for their synergistic regulation of tissue function. Within this framework, macrophages serve as a principal source of ligands that initiate fibrotic programs in fibroblasts, and this regulation may occur through direct cell-to-cell contact (54). The spatial proximity between macrophages and fibroblasts provides a physical basis for these interactions. Spatial analysis of FS tissue by Ng et al. (18) revealed significant co-localization of pro-inflammatory macrophages and fibroblasts in highly fibrotic regions, indicating that macrophages may provide immune activation cues and shape the functional state of fibroblasts. Consistent with these spatial findings, functional studies have demonstrated enhanced migration and proliferation of FS fibroblasts under inflammatory conditions. Moreover, the infiltration of pro-inflammatory macrophages positively correlates with fibroblast activation, likely mediated by macrophage-derived inflammatory factors, such as IL-1β, IL-6, and TNF-α (55) (**Figure 1D**).

In addition to directly secreting pro-inflammatory cytokines, macrophages may indirectly influence the activation process of fibroblasts by modulating other cell types or the extracellular environment (54). For instance, during the early stages of FS, the initial inflammatory signals released by macrophages may facilitate the activation of helper T cell 17 (Th17) subsets. IL-17A, derived from activated Th17 cells, can further induce a transformation towards a pro-fibrotic phenotype by binding to Interleukin-17 receptor A (IL-17RA) on the surface of fibroblasts. Concurrently, IL-17A can also enhance the secretion of various chemokines by fibroblasts, such as CCL2, CCL5, and CCL20, which collectively augment the recruitment of immune cells and sustain the active state of local inflammation (15) (**Figure 1D**).

Among the factors influencing macrophage numbers and immune activation, CCL2 stands out as a particularly noteworthy substance. It is widely acknowledged as a primary ligand in CCR2 chemotaxis, demonstrating significant functional specificity for tissue inflammation and immune cell migration. CCL2 and its receptor, CCR2, effectively mediate the targeted recruitment of monocytes and macrophages, and have been identified in multiple models as a pivotal link in enhancing interactions between macrophages and fibroblasts. Within various monocyte chemotaxis networks, the CCL2/CCR2 axis exhibits a higher mobilization priority, serving as a critical node that drives the spread of inflammation and disease progression. Raghu et al. (56) developed OA models in mice with Ccl2, Ccr2, Ccl5, and Ccr5 gene defects to compare the effects of these signaling axes on monocyte recruitment and pathological evolution of OA. This research indicates that the absence of CCL2 or CCR2 significantly reduces synovitis and macrophage infiltration, thereby alleviating histopathological changes in OA tissue; however, the absence of CCL5 or CCR5 does not yield similar protective effects. Although CCL2 can be produced by various cell types, existing research suggests that mesenchymal-derived cells, particularly fibroblasts, may serve as important initial sources of CCL2 during the early stages of tissue injury. Multiple studies have confirmed that fibroblast-derived CCL2 is essential for the influx of monocytes to the lesion site (56, 57).

Notably, although CCR2 can interact with multiple ligands, including CCL2, CCL7, CCL8, and CCL12, CCL2 has been identified as the primary factor responsible for the mobilization of CCR2^+^ inflammatory monocytes into the bloodstream and sites of injury (58). Additionally, the study found that once these monocytes infiltrate the lesion, they can transform into macrophages that retain the ability to express CCL2. These macrophages sustain their inflammatory nature through autocrine processes, thereby amplifying the recruitment of additional monocytes or fibroblasts and establishing an inflammatory feedback loop centered on CCL2 (59) (**Figure 1D**). Cross-cellular communication between macrophages and fibroblasts via the CCL2/CCR2 axis represents a promising research avenue. However, direct evidence confirming the presence of this CCR2-dependent positive feedback loop in FS is still lacking. Establishing this mechanism would deepen the understanding of the inflammation–fibrosis continuum in FS and provide a conceptual basis for developing targeted therapeutic strategies.

Following the initial mobilization of cells triggered by inflammatory signals, the progression of FS fibrosis often depends on deeper functional interactions between immune and stromal cells. Transforming growth factor (TGF)-β is a central profibrotic signal that amplifies inflammation-driven fibroblast activation and matrix deposition in FS (60). Within the fibrotic microenvironment, macrophages are critical donors of TGF-β, inducing the metabolic reprogramming of adjacent fibroblasts through paracrine signaling and promoting ROS production and ECM synthesis. In turn, fibroblasts secrete and activate TGF-β in an autocrine manner, sustaining their activated phenotype and driving their differentiation into myofibroblasts (18, 61). This reciprocal regulation further enhances the profibrotic function of macrophages, forming a self-reinforcing cycle centered on TGF-β. Clinical and experimental data support this pathological process. Increased expression of TGF-β receptors in patient tissues correlates with disease duration (62), and activation of downstream mothers against decapentaplegic homolog (Smad) 2/3/4 signaling in both macrophages and fibroblasts has been confirmed in multiple FS models (63–66). This bidirectional crosstalk provides a persistent molecular driver of chronic ECM accumulation and shoulder stiffness in FS (**Figure 1D**).

In conclusion, the development and progression of the fibrotic process in FS are intricately linked to dynamic interactions between macrophages and fibroblasts. The reciprocal activation of CCL2, TGF-β, and various inflammatory signals between these cells establishes a self-perpetuating fibrotic network, which constitutes a critical mechanism for the sustained progression of FS.

In addition to macrophages, multiple immune cell populations, including T cells, B cells, mast cells, and their respective subsets, have been identified within the capsular tissues of patients with FS, indicating a highly complex immune microenvironment within the capsule (16, 17, 67). Among these, mast cells frequently exhibit pronounced infiltration in the rotator interval and capsular tissues, where the release of inflammatory mediators may contribute to extracellular matrix remodeling. In parallel, IL-17A derived from Th17 cells enhances the pro-inflammatory and pro-migratory properties of fibroblasts (15, 16).

Although direct experimental evidence demonstrating specific interactions among these immune cell types in FS remains limited, prior studies suggest that macrophages can influence T-cell activation through antigen presentation and cytokine-mediated regulation (15, 68), and may modulate the behavior of other immune subsets through analogous mechanisms (69, 70). Taken together, the available evidence supports the notion that macrophages may occupy a central integrative position within the capsular immune network, coordinating diverse immune signals and transmitting inflammatory cues to fibroblasts. Through this role, macrophages may serve as a critical link between inflammatory amplification and fibrosis progression. Examining the inflammatory–fibrotic evolution of FS from a macrophage-centered perspective may offer a more coherent framework for understanding its complex pathological landscape.

## 3 Macrophage polarization in the pathogenesis of glenohumeral capsule fibrosis

Macrophages are deeply involved in the fibrotic process of FS by shaping fibroblast activation and matrix remodeling; however, their net effects are highly context-dependent and are often discussed in relation to polarization-associated programs. In broad terms, M1-like states tend to align with inflammatory amplification, whereas M2-like states are more frequently associated with immune regulation and tissue repair processes. Importantly, macrophage phenotypes are plastic rather than fixed, and cells can transition between these functional programs to match the needs of different disease phases (71, 72). While the M1 and M2 frameworks serve as a useful foundation for categorizing macrophage functional tendencies, *in vivo* states often extend across a more continuous and multidimensional spectrum that a strict binary classification may not adequately capture. Various disease-associated phenotypes have been identified, including M3, M4, M17, Mreg, Mox, Mhem, and M(hb). In addition to sharing classical M1 or M2 markers, many of these macrophage phenotypes express unique sets of context-specific genes that provide specialized functions tailored to the local tissue needs. These additional programs may enable macrophages to either amplify inflammatory responses for pathogen or tumor clearance or, conversely, to suppress inflammation and aid in the removal of metabolic byproducts and damaged matrix components, thereby contributing to tissue homeostasis (73, 74). This pattern may reflect the plasticity of macrophages, where phenotypes shift in response to local cues and disease stages, rather than remaining fixed. In this context, M1-like and M2-like are better understood as functional orientations, and while marker genes can help stratify states, they may not, on their own, define macrophage function without considering the surrounding microenvironment.

Viewed in this light, the macrophage states revealed by single-cell analyses are more appropriately interpreted as context-dependent deployments of shared functional programs rather than fixed or lineage-distinct entities, thereby suggesting that an M1- and M2-like functional orientation may serve as a pragmatic framework for mechanistic discussions in FS. In FS, single-cell profiling of the capsule has identified macrophage states associated with divergent trajectories, including a MERTK^low^ CD48^+^ population linked to inflammatory activation and a MERTK^+^ LYVE1^+^ MRC1^+^ population enriched with inflammation-restraining and matrix-remodeling features. MERTK-positive macrophages co-express classical repair-associated markers, such as CD163 and CD206, and are also observed in fetal shoulder tissue and remission-phase rheumatoid synovium, suggesting that MERTK expression may mark a conserved pro-resolution macrophage program rather than a disease-specific state. Furthermore, interactions between these macrophages and DKK3^+^ POSTN^+^ fibroblast-like cells have been proposed to facilitate extracellular matrix reorganization and fibrosis regression in FS, potentially contributing to the self-limiting course observed in a subset of patients (18). In the subacromial bursa, macrophages differentiate into regulatory and repair-associated states, which are enriched with CD163, CD206, HSPB1, and F13A1, or more inflammatory or immune-activating states characterized by CD86, PHLDA1, FCER1A, and antigen presentation signatures. Degenerative rotator cuff disease tends to be relatively enriched with the former states, whereas traumatic injury favors the latter, suggesting that distinct macrophage programs may influence divergent inflammatory trajectories in shoulder pathology (75).

Current evidence indicates that macrophage phenotypes exist along a continuum influenced by local microenvironmental cues rather than fixed identities. While single-cell analyses have refined the classification of macrophage phenotypes, many identified subsets retain core functional features that broadly align with either inflammatory or repair-associated programs. Given the limited direct evidence on macrophage polarization in FS, this section refers to conserved mechanisms described in other fibrotic conditions to aid in the interpretation. Accordingly, in this review, M1-like and M2-like are used as functional orientations to characterize macrophage behavior in FS, with selected subsets discussed when they offer disease-relevant mechanistic insights into the coupling of inflammation and fibrosis.

**Figure 2:**
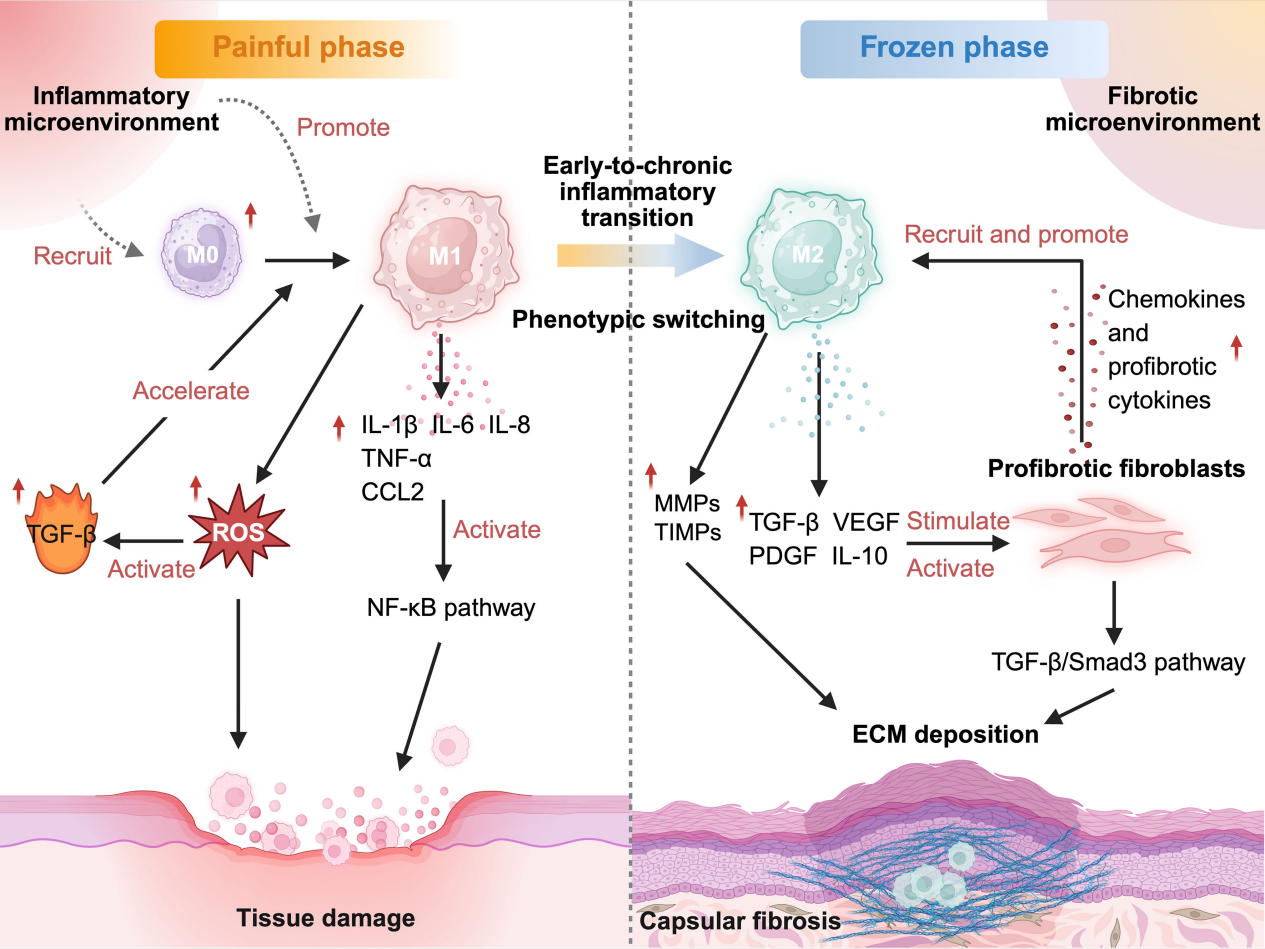
Dynamic microenvironmental transitions shape macrophage phenotypes and drive fibrotic remodeling in FS. During the inflammatory phase, inflammatory cues recruit monocytes and promote their differentiation to M1-like macrophages. These macrophages amplify inflammation and oxidative stress through NF-κB and ROS signaling, enhancing TGF-β activation and accelerating capsular injury. Unresolved inflammation progresses to a chronic state, fostering a fibrotic environment that promotes M2-like polarization. M2-like macrophages dysregulate MMP/TIMP-mediated matrix turnover and sustain TGF-β/Smad3 signaling, leading to persistent fibroblast activation and enhanced ECM accumulation. Chemokines and profibrotic cytokines released by activated fibroblasts establish a feedback circuit that recruits and maintains M2-like macrophages, thereby reinforcing long-term capsular fibrosis. *Created in BioRender. Liang, H. (2025)**https://BioRender.com/gnka1qm*. FS, frozen shoulder; IL-1β, interleukin-1 beta; IL-6, interleukin-6; IL-8, interleukin-8; TNF-α, tumor necrosis factor alpha; CCL2, C-C motif chemokine ligand 2; TGF-β, transforming growth factor beta; ROS, reactive oxygen species; NF-κB, nuclear factor kappa-light-chain-enhancer of activated B cells; MMPs, matrix metalloproteinases; TIMPs, tissue inhibitors of metalloproteinases; VEGF, vascular endothelial growth factor; PDGF, platelet-derived growth factor; IL-10, interleukin-10; Smad3, mothers against decapentaplegic homolog 3; ECM, extracellular matrix.

As fully demonstrated, M1-like macrophages are the central force driving the onset and escalation of tissue inflammation, leading to tissue damage by perpetuating the inflammatory process (76, 77). Within the capsule tissue of FS, there is a notable increase in M0 macrophages, which primarily differentiate from monocytes recruited in the early phases of inflammation and tend to polarize into the M1-like phenotype (30). Gene enrichment analysis has shown that the differentially expressed genes linked to these cells are significantly enriched in various biological processes, particularly those involving cytokine receptor interactions, collagen metabolism regulation, and inflammatory cascade reactions (17). M1-like macrophages release inflammatory factors, such as TNF-α, IL-1β, and IL-6, which can induce cytotoxicity, worsen tissue damage, and facilitate fibrosis progression by activating the NF-κB pathway (78, 79). Furthermore, in a fibrotic environment, ROS released by M1-like macrophages not only directly harm parenchymal cells but also promote the activation of latent TGF-β. This activated TGF-β enhances the pro-inflammatory response of macrophages and sets the stage for subsequent pro-fibrotic signals (80) (**Figure 2**).

While the pro-inflammatory role of M1-like macrophages during the onset of inflammation is well-established, the involvement of M2-like macrophages in fibrosis is more intricate and remains a subject of debate. From a functional perspective, within the broadly defined M2-like orientation, M2 macrophages comprise multiple phenotypically and mechanistically distinct subsets, rather than a single homogeneous population. Based on their inducing signals, M2 macrophages are commonly categorized into M2a, M2b, M2c, and M2d subtypes, each characterized by distinct marker expression and cytokine secretion profiles that translate into nuanced immunoregulatory behaviors (81). Among these subsets, IL-4- or IL-13-induced M2a macrophages exhibit a secretory profile enriched in IL-10, TGF-β, and Th2-associated chemokines and produce matrix-related mediators, such as fibronectin, linking M2a polarization to enhanced tissue repair, ECM synthesis, and fibrosis-associated programs across multiple disease models (82, 83). In contrast, M2c macrophages respond to IL-10 or glucocorticoid signaling and display potent anti-inflammatory and immunosuppressive properties. By suppressing pro-inflammatory cytokines and promoting matrix degradation and clearance, M2c macrophages contribute to the restoration of tissue homeostasis and may exert protective effects against maladaptive fibrosis (82, 84). Although M2b and M2d subsets are also involved in immune regulation, accumulating evidence suggests that the balance between fibrosis-promoting M2a programs and resolution-oriented M2c responses critically influences whether tissue remodeling proceeds toward effective resolution or pathological collagen deposition (85–87). Nonetheless, the presence and functional delineation of these M2 subsets in FS remain to be conclusively demonstrated, and additional evidence is required to define their roles within the capsular microenvironment.

In the context of FS, M2-like macrophages appear to exhibit a comparable functional duality in immune regulation and tissue remodeling, which may be interpreted in light of the functional heterogeneity described above. Under appropriate microenvironmental conditions, M2-like macrophages secrete regulatory and reparative mediators that attenuate inflammation and support tissue homeostasis restoration. However, when these reparative programs become persistently activated or dysregulated, the same pathways may shift toward excessive collagen synthesis, thereby promoting maladaptive capsule remodeling and fibrosis.

Some research suggests that M2-like macrophages may contribute to anti-inflammatory responses and help maintain homeostasis during certain phases of FS. Transcriptome data revealed a significant upregulation of M2-related genes (such as Il10, Cd14, and Cd163) in the tissues of patients with FS, while M1-related factors (such as TNF-α, IL-6, and IL-8) and their downstream NF-κB signaling exhibited an inhibitory trend, suggesting a gradual weakening of local inflammatory activity (75, 88). Further research has shown that although fibroblast activation markers in FS tissue are continuously upregulated, the expression of the inflammatory marker CD90 decreases, indicating that fibroblasts may have adopted a repair-oriented activation phenotype (88). This transition is consistent with the polarization state of macrophages, where the M2 subset, especially M2c, contributes to reducing the prolonged stimulation of fibroblasts. These clusters exert this effect by inhibiting the production of pro-inflammatory cytokines, thereby curtailing excessive fibroblast activation and collagen deposition (89, 90). Additionally, as phagocytic cells with clearance functions, M2-like macrophages can directly participate in ECM clearance by engulfing excess collagen, which aids in alleviating matrix barrier abnormalities (80). These findings suggest that at specific stages of FS, M2-like macrophages may exert protective effects through mechanisms such as anti-inflammatory action, repair, and clearance, and their increased proportion may help counteract M1-mediated inflammatory chain reaction.

However, numerous studies have identified M2-like macrophages as the primary agents responsible for exacerbating fibrosis. In FS-related pathology, clinical evidence suggests a tendency for macrophages to skew toward M2-like functional program. Serum from patients who developed secondary FS after rotator cuff repair polarized human macrophages toward an M2-oriented phenotype rather than M1, marked by increased expression of Arginase-1 (Arg-1), TGF-β, and IL-10. This M2-biased polarization is accompanied by enhanced activation of capsule-derived fibroblasts, indicating that M2-like macrophage-driven immunoregulatory programs may contribute to fibrotic formation within the glenohumeral capsule (66). Similarly, single-cell transcriptomic analysis of human osteoarthritic synovium revealed increased infiltration of macrophages enriched for the expression of genes such as CD163, MRC1, C1QC, APOE, and F13A1, along with a higher proportion of M2-polarized macrophages than in non-OA controls. These macrophages exhibited transcriptional programs associated with extracellular matrix organization and synovial remodeling and displayed extensive interactions with fibroblasts, implicating M2-like macrophage states in synovial fibrotic remodeling (91).

Consistent with these observations, evidence from diverse fibrotic disease models supports the crucial role of M2-like macrophage programs in orchestrating matrix remodeling and fibroblast activation. M2-like macrophages are a major source of profibrotic mediators, including TGF-β, platelet-derived growth factor (PDGF), and vascular endothelial growth factor (VEGF), which directly induce fibroblast activation and ECM deposition (21, 92). They also produce MMPs and TIMPs, the MMP/TIMP balance is essential for tissue homeostasis, whereas TIMP overexpression restricts matrix degradation and excessive MMP activity disrupts tissue architecture, both favoring maladaptive repair and collagen accumulation (93, 94). Within this program, TGF-β functions as a central signaling node, guiding the shift from repair to fibrosis. M2-like polarization is frequently accompanied by enhanced TGF-β/Smad3 signaling, which drives fibroblast phenotypic conversion and augments collagen deposition (95). Notably, this regulation appears to be bidirectional: M2-derived growth factors activate fibroblasts, and activated fibroblasts upregulate fibrosis-associated molecules, such as fibroblast activation protein (FAP) and chemokines, which reinforce M2-like polarization, establishing a self-amplifying circuit. Nevertheless, confirmation of this pathway in FS is still required (96) (**Figure 2**).

The shift from repair signals to pro-fibrotic signals illustrates the dynamic remodeling of macrophage polarization as FS progresses. Macrophage plasticity results in differentiation at various stages. In the early stage of FS marked by pain hypersensitivity, macrophage populations tend to adopt M1-like functional programs, which are associated with elevated inflammatory activity (15, 17, 32) As the disease progresses and capsular stiffness becomes more apparent, M2-like macrophages emerge as the dominant population. Evidence from FS-related studies indicates that patients experiencing secondary FS after rotator cuff repair exhibit more pronounced shoulder stiffness and limited ROM. Additionally, serum-derived factors from these patients can induce M2-like polarization of THP-1 macrophages while simultaneously activating capsule-derived fibroblasts via the TGF-β/Smad3 pathway, promoting capsular thickening and fibrotic remodeling (17, 66).

Consistent with the pathological features of FS, including fibroblast proliferation, synovial thickening, ECM remodeling, and persistent low-grade inflammation (1), these observations support the notion that a transition toward M2-like macrophage dominance may occur during the frozen stage. Although M2-like macrophages are essential for tissue repair, sustained activation of their reparative programs under chronic inflammatory conditions may inadvertently promote excessive collagen deposition, ultimately contributing to capsular fibrosis (**Figure 2**).

M1-like and M2-like macrophages are not mutually exclusive during fibrosis; they frequently coexist within the same lesion, with their relative abundance determined by the disease stage and local microenvironment. Cross-disease studies support this dynamic pattern. M1-like macrophages amplify early inflammation and tissue injury through factors such as TNF-α, IL-1β, IL-6, and CCL2, indirectly promoting fibroblast activation and migration. In contrast, M2-like macrophages drive fibroblast differentiation and directly stimulate ECM deposition by upregulating TGF-β, IL-10, and TIMPs (93, 97, 98). Time-resolved analyses have demonstrated that M1-like macrophages predominate during the early inflammatory phase and gradually give way to M2-like macrophages, a transition closely correlated with increased collagen deposition and fibrotic remodeling (99). Consistent temporal patterns have been observed in myocardial injury models, where M1-like macrophage dominance peaks at approximately day 3 post-injury, followed by an expansion of M2-like macrophages around day 7, coordinating tissue repair and fibrosis (100). Notably, epigenetic regulatory mechanisms may shape the temporal relationship between inflammation and fibrosis. In a spinal cord injury model characterized by significant neuroinflammation and progressive fibrotic or scar-forming changes, circRNA CDR1as was observed to regulate inflammatory and fibrotic responses in a time-dependent manner. This regulation occurs through microglial modulation, activation of the TGF-β/Smads signaling pathway, and progressive accumulation of ECM in the later stages. Together, these observations indicate that immune-to-fibrotic transitions may not be restricted to peripheral tissues (101, 102). Nevertheless, despite these converging lines of evidence, the direct temporal and programmatic characterization of macrophage polarization dynamics in FS remains limited. Consequently, further investigation is needed to understand the precise timing, regulatory cues, and functional transitions of macrophage subsets across different FS stages.

In summary, fibrosis in FS is unlikely to be driven by a single macrophage population but rather reflects the stage-dependent interplay between M1-like and M2-like subsets. Temporal shifts in their polarization states shape the transition from inflammation to fibrosis, underscoring the importance of understanding the coordinated dynamics of disease progression. Current temporal evidence for FS remains limited, highlighting the need for a more systematic characterization of this process.

## 4 CCL2/CCR2 signaling axis mediates macrophage recruitment and polarization dynamics

In the previous section, we highlighted the significance of the CCL2/CCR2 signaling axis in facilitating intercellular communication. Beyond its initial role, this axis is instrumental in recruiting inflammatory monocytes and macrophages, as well as their subsequent polarization. For effective polarization, a sufficient pool of macrophages is necessary; hence, early recruitment driven by CCL2/CCR2 focuses on CCR2^+^ monocytes at the lesion site, thereby expanding the local macrophage population and setting the stage for ensuing differentiation. This section delves into the dual role of the axis in macrophage polarization. Initially, CCL2 aids in the influx of CCR2^+^ cells, creating a conducive environment for inflammatory processes. Subsequently, downstream signals linked to this pathway can influence polarization programs in various directions, affecting local inflammation and fibrosis. Unraveling the mechanisms that guide these pathways will be crucial for pinpointing immune control points in FS progression.

**Figure 3:**
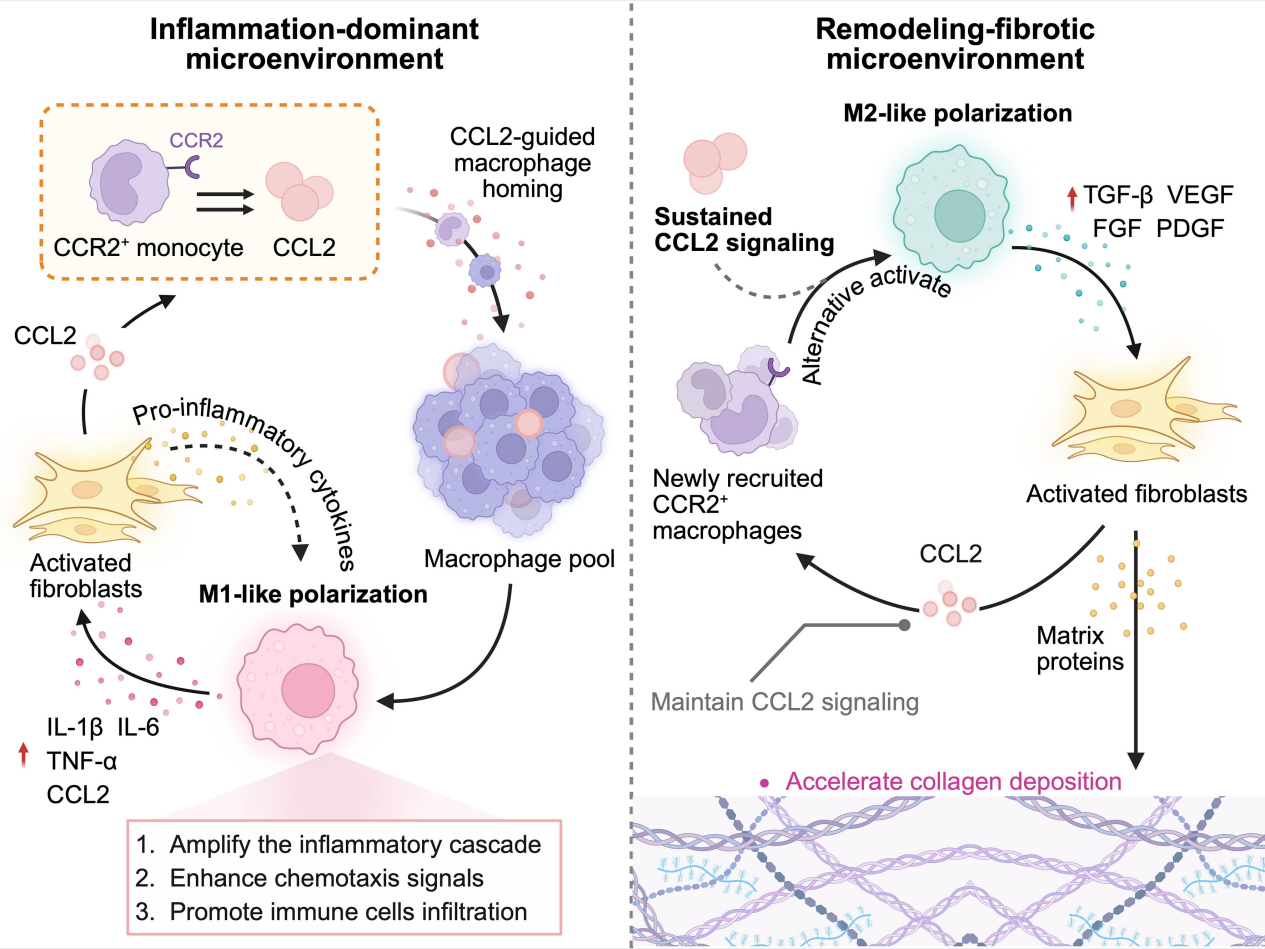
CCL2/CCR2 signaling facilitates the acquisition of distinct macrophage phenotypes across various microenvironmental contexts in FS. In an inflammation-dominant setting, elevated CCL2 attracts CCR2^+^ monocytes into the capsule, thereby expanding the macrophage pool. Under persistent stimuli, these cells polarize toward M1, release inflammatory mediators, activate fibroblasts, and sustain an inflammatory milieu. Continued CCL2/CCR2 activity maintains recruitment, while alternative cues increasingly favor M2-like polarization, forming the cellular basis of fibrosis. The M2-like macrophages secrete growth factors that further activate fibroblasts and drive excessive matrix production. Activated fibroblasts produce CCL2, reinforcing CCL2/CCR2 signaling and stabilizing profibrotic macrophage programs. *Created in BioRender. Liang, H. (2025)**https://BioRender.com/i1032at*. FS, frozen shoulder; CCL2, C-C motif chemokine ligand 2; CCR2, C-C motif chemokine receptor 2; IL-1β, interleukin-1 beta; IL-6, interleukin-6; TNF-α, tumor necrosis factor alpha; TGF-β, transforming growth factor beta; VEGF, vascular endothelial growth factor; FGF, fibroblast growth factor; PDGF, platelet-derived growth factor.

In the initial phase of injury, CCL2 is instrumental in attracting a significant influx of CCR2 positive monocytes and macrophages to the lesions, promoting their polarization into the M1 phenotype and triggering an inflammatory response. These populations subsequently release pro-inflammatory factors, such as TNF-α and IL-1β, which further enhance CCL2 expression, thereby reinforcing the inflammatory cycle. They also facilitate the activation of fibroblasts and the expression of fibrosis markers, including α-SMA and vimentin (103). Once activated, inflammatory fibroblasts can produce CCL2, which further intensifies the local CCL2/CCR2 axis activity and cell communication density (104) (**Figure 3**). As CCL2/CCR2 signaling persists, some macrophages begin to undergo alternative activation (M2 polarization), marked by increased levels of TGF-β, chitinase-like protein 3, and arginase 1, which are classic indicators of M2 macrophage activation, indicating a shift towards tissue remodeling (105). Notably, the ongoing release of CCL2 by M1 macrophages not only sustains the inflammatory state but also draws a new wave of inflammatory monocytes and immature macrophages to the lesion via CCR2-mediated signaling. In the context of the chronic injury microenvironment, these newly recruited cells are more likely to receive alternative activation signals and adopt the M2 phenotype upon entering the tissue, releasing substantial amounts of pro-repair signals, such as TGF-β1, which continuously stimulate fibroblast activation and exacerbate tissue fibrosis (100) (**Figure 3**).

Intriguingly, CCL2 appears to have the capacity to simultaneously promote the polarization of both M1 and M2 macrophages, a phenomenon likely due to its dual role as a “resource supplier” and an “effect amplifier.” On the one hand, driven by inflammatory signals, CCL2 preferentially mobilizes Ly6C^+^ CCR2^+^ inflammatory monocytes through CCR2-mediated signaling, thereby determining the size of the polarized macrophage pool. In contrast, even the absence of the CX3C motif chemokine receptor 1 (CX3CR1), which is also highly expressed on macrophage surfaces, does not significantly impact this recruitment process, underscoring the specific role of CCR2 as a core receptor in the ligand-receptor axis (106). From another perspective, the direction of macrophage polarization is primarily influenced by the tissue microenvironment, which is highly complex and dynamic in disease states. Thus, CCL2 is more likely to function as a signal amplifier rather than a sole driving factor, maintaining existing signals while enhancing the polarization trends in specific environments. This regulatory feature may explain why CCL2/CCR2 exhibits markedly different macrophage polarization induction effects across various pathological contexts. For instance, in a chronic obstructive pulmonary disease model, CCL2 overexpression recruits and activates macrophages via the PI3K/AKT pathway, leading to the concurrent upregulation of M1 and M2 markers, ultimately driving chronic inflammation and airway remodeling (107). However, in the context of myocardial infarction, CCL2 overexpression significantly promotes M1 and inhibits M2 polarization by activating the p38 mitogen-activated protein kinase (p38 MAPK) and NF-κB signaling pathways, resulting in a sustained immune response and excessive collagen deposition, thereby exacerbating cardiac remodeling (108).

These differences underscore how the CCL2/CCR2 signaling axis shapes tissue repair paths through context-dependent regulation of the local microenvironment and immune dynamics. The fibrotic outcome ultimately depends on the functional reprogramming of macrophages at different stages and the persistence of their recruitment. In this setting, a single biomarker is insufficient to predict disease progression; reliable assessment requires the integration of phenotypic signatures with functional programs (109). Notably, the sustained dominance of any macrophage phenotype can disrupt tissue homeostasis, with persistent CCL2/CCR2 signaling potentially amplifying this imbalance. Rather than dictating a fixed polarization fate, this axis primarily modulates the inflammatory–repair equilibrium by expanding the recruitment of inflammatory cells and reinforcing existing microenvironmental cues.

The mechanisms involved have significant implications for the pathogenesis of FS. The presence of both inflammatory signals and fibrotic responses in FS lesion tissues indicates that CCL2 may not only facilitate monocyte recruitment via CCR2 and increase the local pool of inflammatory cells, but also enhance pro-inflammatory or pro-repair/fibrotic tendencies, depending on the specific signaling context. In a highly inflammatory environment, CCL2/CCR2 may sustain M1 dominance, resulting in chronic inflammation and tissue injury. Conversely, when repair signals prevail, this axis may shape the functional orientation of M2 macrophages to adapt to the shifting microenvironment, with fibrosis potentially arising as a maladaptive consequence (**Figure 3**). Understanding this context dependency is crucial for elucidating the intertwined mechanisms of inflammation and fibrosis in FS, as well as for developing therapeutic strategies targeting the CCL2/CCR2 axis.

## 5 Mechanical cues shape macrophage function and fibrotic remodeling

**Figure 4:**
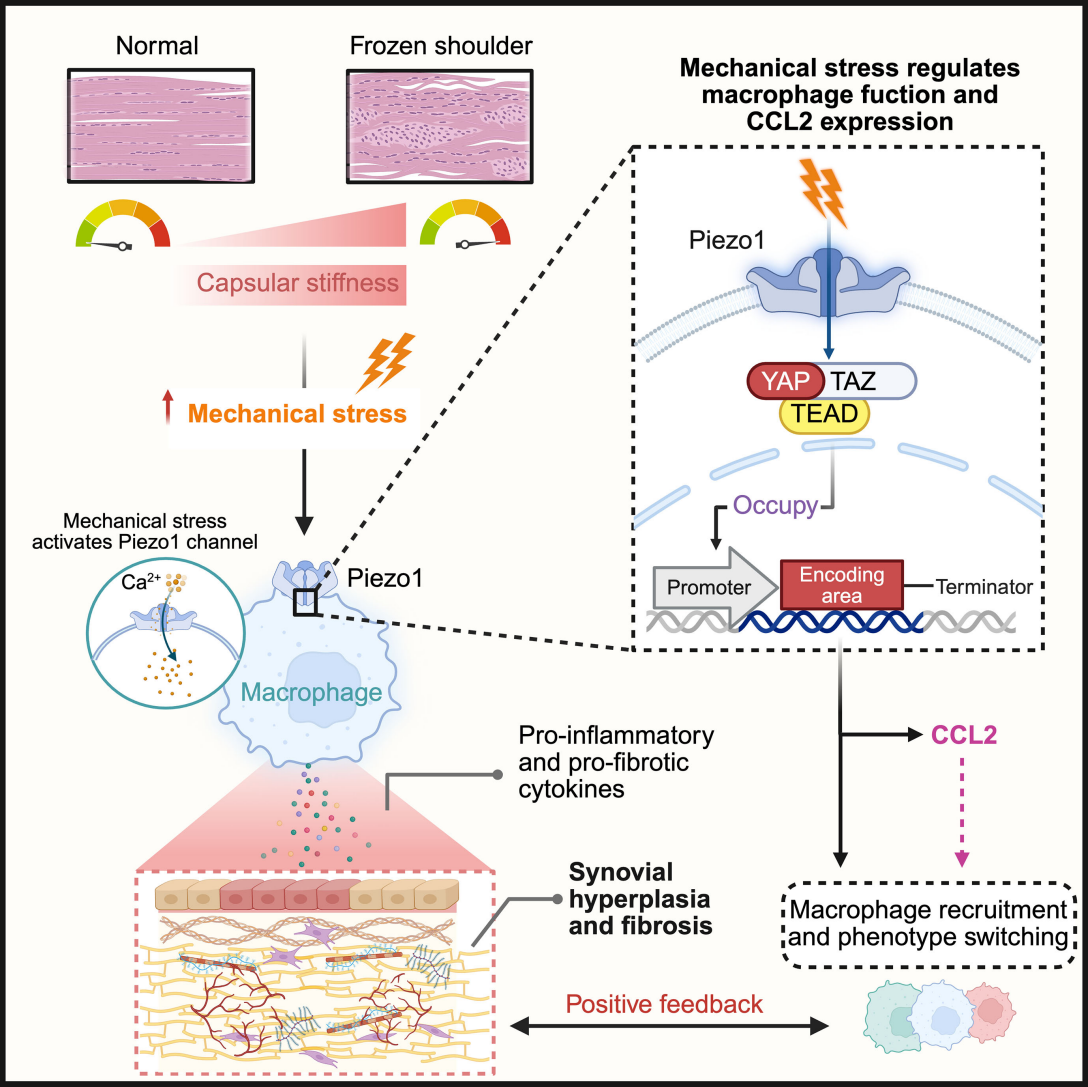
Mechanical stress regulates macrophage function via Piezo1 to facilitate fibrotic progression in FS. In FS, the capsule demonstrates increased stiffness, thereby augmenting local mechanical stress. This increased mechanical loading activates the mechanosensitive ion channel Piezo1 in macrophages, resulting in Ca²^+^ influx, which triggers altered expression patterns of pro-inflammatory and pro-fibrotic mediators, exacerbating synovial thickening and fibrosis. Following Piezo1 activation, the transcriptional co-regulators YAP and TAZ form a complex with TEAD and translocate into the nucleus. This complex binds to promoter regions to regulate gene transcription, thereby driving macrophage functional reprogramming and upregulating CCL2 expression. The sustained activation of this mechanotransduction pathway significantly influences macrophage behavior and function, establishing a positive feedback circuit that accelerates the formation of capsular fibrosis. *Created in BioRender. Liang, H. (2025)**https://BioRender.com/f0qil6v*. FS, frozen shoulder; CCL2, C-C motif chemokine ligand 2; Piezo1, Piezoelectric ion channel 1; YAP, yes-associated protein; TAZ, transcriptional coactivator with PDZ-binding motif; TEAD, TEA domain transcription factor; Ca²^+^, calcium ion.

Mechanical stress is a fundamental physical cue through which cells sense and respond to their microenvironment, and mechanotransduction critically regulates inflammation, hyperplasia, and fibrotic remodeling following tissue injury (110). The glenohumeral capsule is an extracellular matrix–dominated dense fibrous connective tissue primarily composed of type I and type III collagen, with collagen fibers arranged in a multi-axial orientation to accommodate multidirectional joint loading (9, 111, 112). Based on these intrinsic mechanical characteristics of the capsule, accumulating evidence indicates that pathological alterations in ECM organization and stiffness are closely linked to the progression of FS. A study constructing a three-dimensional model of the glenohumeral joint based on CT arthrography demonstrated that the capsular tissue in FS exhibits increased stiffness and a persistently high-tension state, reflecting a mechanically imbalanced intracapsular environment (113). This mechanical dysregulation is largely attributable to the excessive accumulation of ECM together with enhanced collagen cross-linking, which progressively disrupts the physiological mechanical balance of the capsule, leading to restricted glenohumeral mobility and driving disease progression (**Figure 4**) (13, 112).

Macrophages represent a significant subset of infiltrating immune cells in FS and are increasingly acknowledged as potential sensors of stiffness-related mechanical cues within the capsule microenvironment. Emerging evidence suggests that macrophages can detect matrix stiffness and mechanical forces, resulting in functional reprogramming toward inflammatory or pro-fibrotic states. *In vitro*, Meizlish et al. (114) demonstrated that IL-4–polarized bone marrow–derived macrophages perceive substrate stiffness and selectively modify the transcriptional programs associated with tissue repair in response to the mechanical state of the ECM. Similarly, Chen et al. (115) reported that substrate stiffness dynamically influences macrophage polarization, with softer matrices favoring an M1-like pro-inflammatory phenotype and intermediate to higher stiffness promoting an M2-like repair-oriented phenotype. Progressive matrix stiffening further enhances M2-associated markers, indicating that a stiff ECM may bias macrophages toward a profibrotic polarization state, thereby reinforcing matrix stiffening (116). Within the musculoskeletal system, changes in physiological tendon stiffness have been shown to affect the proliferation and morphology of human tendon-derived cells and alter the cytokine secretion profile and functional behavior of THP-1–derived macrophages in ways that may favor tendinopathy development (117). Thus, although these models do not fully recapitulate the complexity of the *in vivo* microenvironment, they are largely based on rodent bone marrow–derived macrophages or THP-1–derived human macrophages, which retain conserved mechanosensitive signaling properties, and therefore provide a reasonable stiffness-dependent mechanistic reference for capsular fibrosis (**Figure 4**). To integrate these observations across disease contexts and experimental systems, the major mechanotransduction-associated immune and fibrotic responses are summarized in **Table 1**.

**Table 1:** Mechanistic evidence for mechanotransduction-associated immune and fibrotic responses derived from frozen shoulder and related disease models.

Main mechanistic findings	Diseases/models	Sample sources	Experimental setup	References
Alterations in ECM stiffness affect the mechanical properties of the glenohumeral capsule	Shoulder instability	Humans	*In vitro*	(112)
	Frozen shoulder	Humans	In silico	(113)
The phenotype and function of macrophages are dynamically regulated in response to variations in matrix stiffness	3D stiffness-tunable hydrogel model, pulmonary fibrosis	Mice	*In vitro*, *in vivo*	(114)
	Stiffness-tunable polyacrylamide hydrogel model, subcutaneous implant model	Mice	*In vitro*, *in vivo*	(115)
	Stiffness-tunable hydrogel model, hepatocellular carcinoma	Humans, Rats	*In vitro*, *in vivo*	(116)
Matrix stiffness–dependent macrophage phenotypic switching may contribute to tendinopathy progression	Stiffness-tunable hydrogel model	Humans	*In vitro*	(117)
Piezo1 functions as a crucial mechanosensor mediating tendon cell responses to mechanical loading	Genetic manipulation of *Piezo1* in tendon-derived cells	Humans, Rats, Mice	*In vitro*, *in vivo*	(119)
Piezo1 orchestrates immune activation and tissue remodeling via mechanotransductive signaling	Acute lung inflammation, pulmonary fibrosis	Humans, Mice	*In vitro*, *in vivo*	(120)
	Bacterial lung infection, pulmonary fibrosis	Mice	*In vitro*, *in vivo*	(121)
	Renal fibrosis	Humans, Mice	*In vitro*, *in vivo*	(122)
	Liver fibrosis	Humans, Mice	*In vitro*, *in vivo*	(123)
	Temporomandibular joint arthritis	Humans, Mice, Rats	*In vitro*, *in vivo*	(124)
	Osteoarthritis	Rats	*In vivo*	(127)
	Osteoarthritis	Rats	*In vitro*, *in vivo*	(128)
Piezo1 links mechanical stress to macrophage-driven fibrosis	Implant-associated fibrosis	Mice	*In vitro*, *in vivo*	(125)
	Temporomandibular joint arthritis	Mice	*In vitro*, *in vivo*	(129)
YAP signaling–driven mechanotransduction enhances inflammation-related fibrotic remodeling, which is closely linked to the functional reprogramming of macrophages	Rheumatoid arthritis	Humans, Mice	*In vitro*, *in vivo*	(133)
	Osteoarthritis	Rats	*In vitro*, *in vivo*	(137)
	Chronic shoulder inflammation	Humans	*In vitro*	(138)
	Implant-associated fibrosis	Mice	*In vitro*, *in vivo*	(134)
	Pulmonary fibrosis	Humans, Mice	*In vitro*, *in vivo*	(135)
	Renal fibrosis	Mice	*In vitro*, *in vivo*	(136)
Piezo1/YAP mechanotransduction cascade regulates the CCL2/CCR2 axis to drive immune cell recruitment and tissue remodeling	Osteoarthritis	Rats	*In vivo*	(127)
	Osteoarthritis	Mice	*In vivo*	(139)
	Pulmonary fibrosis	Humans, Mice	*In vitro*, *in vivo*	(135)
	Cardiac fibrosis	Rats, Mice	*In vitro*, *in vivo*	(140)
YAP-mediated transcriptional regulation of CC chemokines during pathological matrix remodeling	Cardiac fibrosis	Rats, Mice	*In vitro*, *in vivo*	(140)
	Lumbar degeneration	Humans, Mice	*In vitro*, *in vivo*	(141)

Macrophages possess the ability to actively interpret variations in matrix stiffness, raising a critical mechanistic inquiry regarding the manner in which these cells translate external mechanical stimuli into intracellular signaling pathways. Emerging evidence suggests that this conversion process relies on specialized mechanotransduction mechanisms, with mechanosensitive ion channels serving as the primary structural and functional components that facilitate the detection and transduction of mechanical forces by cells (118). Mechanosensitive ion channels, notably Piezo1 and Piezo2, are known to directly respond to mechanical stimuli, such as tension and shear stress, facilitating Ca²^+^ influx and initiating signal transduction pathways (118). Among these channels, Piezo1 has been identified as the primary and functionally specific mechanotransducer in load-bearing connective tissues. Evidence from human tendon cells and corresponding mouse models indicates that mechanical stress–induced Ca²^+^ signaling is predominantly dependent on Piezo1, whereas other mechanosensitive channels, such as transient receptor potential vanilloid 4 (TRPV4) and polycystin-2 (PKD2), appear to play limited roles in this response (119).

Beyond its role in structural mechanosensing within matrix-resident cells, Piezo1 activation plays a crucial role in regulating key immune functions, such as migration, inflammatory activation, and phenotype reprogramming in macrophages, dendritic cells, and T cells (120, 121). Through these mechanisms, Piezo1 links mechanical cues to downstream immune responses that are vital for tissue repair and regeneration (122, 123). Furthermore, a recent study revealed that Piezo1 coordinates interactions between mesenchymal stromal cells and Th17 cells to regulate bone metabolism in a murine temporomandibular joint arthritis model (124), underscoring its broader role as a mechanosensitive integrator between the stromal and immune compartments.

At the macrophage level, mechanistic investigations have elucidated that Piezo1 functions as the principal sensor of stiffness, linking matrix rigidity to macrophage polarization. It demonstrates significantly elevated expression and Ca²^+^ signal transduction capacity compared to Piezo2. Enhanced substrate stiffness activates Piezo1-dependent Ca²^+^ signaling, which subsequently facilitates the activation of macrophage inflammatory programs. Conversely, deletion of *Piezo1* specifically in myeloid cells suppresses pro-inflammatory macrophage polarization, augments the production of anti-inflammatory mediators, and significantly diminishes immune infiltration and fibrotic capsule formation around rigid subcutaneous implants (125). Current evidence indicates that mechanical overload plays a crucial role in driving inflammation and structural remodeling of synovial joints, such as the knee and shoulder, with Piezo1-mediated mechanotransduction serving as the central regulatory mechanism in this process (126). In rat models of osteoarthritis, increased Piezo1 expression has been found to be closely associated with synovial inflammation and fibrotic remodeling (127, 128). Additionally, Shen et al. (129) demonstrated in a murine model of temporomandibular joint arthritis that an imbalance between M1-like and M2-like macrophage populations plays a critical role in synovial inflammation and fibrosis, with this polarization disequilibrium being dynamically regulated by Piezo1 signaling. Collectively, these findings suggest that Piezo1-mediated mechanotransduction links abnormal mechanical cues to immune activation fibrotic diseases. Currently, there is no direct evidence demonstrating Piezo1 activation in macrophages within FS, either in patient-derived tissues or in preclinical models, which limits our understanding of how mechanical signals shape the progression and clinical outcomes of this disease. However, considering the established role of Piezo1 in various fibrotic disorders and the unique mechanical properties of the glenohumeral capsule, it is reasonable to hypothesize that FS involves similar mechanosensitive processes (**Figure 4**). Future research examining the role of Piezo1-driven mechanotransduction within the specific mechanical environment of the glenohumeral capsule could further substantiate the applicability of this hypothesis in FS.

Piezo1-mediated Ca²^+^ influx represents the initial phase of macrophage mechanical perception, with the subsequent activation of transcriptional regulatory modules playing a significant role. The yes-associated protein (YAP) and transcriptional coactivator with PDZ-binding motif (TAZ) are known for tension sensing and gene expression reprogramming across cell types, acting as mechanotransduction transcription factor axes (130). YAP and TAZ are homologous transcription coactivators that operate in the nucleus upon activation. In response to mechanical stimuli, such as increased ECM stiffness or stress overload, YAP/TAZ translocates into the nucleus and forms transcriptional complexes with TEA domain transcription factors (TEAD), thereby governing the expression of genes involved in cell proliferation, differentiation, and extracellular matrix remodeling (131, 132). Through these downstream transcriptional programs, YAP/TAZ modulates synovial cell functions and drives arthritic pathological progression characterized by synovial hyperplasia, pannus formation, immune cell infiltration, and ultimately synovial fibrosis (130, 133). In line with this concept, *in vitro* substrates with adjustable stiffness have demonstrated that variations in matrix rigidity modulate Piezo1 expression and YAP/TAZ activation in macrophages. This modulation subsequently influences whether macrophages predominantly assume M1- or M2-like polarization states (134). *In vivo* models of visceral fibrosis have demonstrated that targeting YAP/TAZ signaling in macrophages can shift them between pro-inflammatory and pro-fibrotic states, thereby affecting the severity of fibrosis. This indicates that YAP/TAZ plays a crucial role in determining macrophage phenotype in fibrotic tissues (135, 136). In the musculoskeletal system, Li et al. (137) demonstrated that activating the YAP axis in skeletal stem cells using microgel-based carriers enhances their immunomodulatory reprogramming. This, in turn, modulates macrophage polarization and alleviates osteoarthritic pathology. While direct evidence of a similar YAP/TAZ-mediated mechanism in FS is still lacking, a recent study on fibroblast-like cells derived from the subacromial bursa of patients with chronic shoulder disease revealed that these cells exhibit YAP-dependent mechano-responsiveness. Increased cyclic loading promotes YAP nuclear localization, disrupts the MMP/TIMP balance, and drives ECM production and tissue remodeling (138). Collectively, these findings suggest that YAP/TAZ-driven mechanotransduction is relatively conserved across fibrotic contexts. Its ability to reprogram macrophages and stromal cell phenotypes may offer a valuable framework for understanding the mechanically driven inflammation-fibrosis coupling in FS, warranting further investigation.

Piezo1 activation not only directly influences macrophage function but is also intricately involved in modulating chemotactic signaling pathways. The process of mechanical loading is linked to the upregulation of CCL2 and CCR2 expression, which appears to be contingent on Piezo1 activation. In rodent models of osteoarthritis, transcriptomic profiling of joint tissues has indicated a persistent upregulation of several genes implicated in inflammatory responses and mechanosensing, such as *Ccl2* and *Piezo1*. This is accompanied by a significant enrichment of gene programs associated with synovial hyperplasia and fibrosis within the synovium (127, 139). These findings suggest that Piezo1-mediated mechanosignaling may be crucial for regulating the CCL2/CCR2 axis in response to mechanical stress.

Although Piezo1-mediated mechanosignaling is closely associated with the expression of CCL2, it does not appear to directly regulate CCL2 or CCR2 transcription, implying the involvement of additional intermediary regulators. In pulmonary fibrosis models, YAP/TAZ activation results in increased CCL2/CCR2 expression, facilitating the recruitment of monocytes and macrophages, thereby intensifying inflammatory infiltration and promoting fibrosis. Remarkably, neutralizing CCL2 has been shown to reverse these effects, indicating that the development of pulmonary fibrosis driven by YAP/TAZ is at least partially dependent on the CCL2/CCR2 axis (135). This phenomenon was consistently observed in a cardiac fibrosis model. Subsequent mechanistic analyses demonstrated that YAP/TAZ can bind to the *Ccl2* promoter, thereby directly enhancing its transcription (140). This finding contributes to a deeper understanding of the mechanism by which CCL2 responds to mechanical cues (**Figure 4**). Interestingly, a study reported that under lumbar instability, abnormal mechanical stress interferes with YAP/TEAD signaling, thereby reducing YAP activity in the cartilage endplate cells. This interference results in increased CCL3 expression, which recruits and activates osteoclasts originating from bone marrow monocytes, leading to pathological remodeling of the endplate. Mechanistically, YAP/TEAD complexes consistently function by occupying the *Ccl3* promoter region and regulating its transcriptional activity (141). Although these studies focused on different cellular processes and produced tissue-specific effects, they both highlighted that YAP-dependent regulation of CC chemokine transcription is a conserved mechanotransductive mechanism. This mechanism allows mechanical stress to direct immune cell recruitment and target tissue remodeling in a context-dependent manner.

In summary, the Piezo1–YAP/TAZ–CCL2/CCR2 signaling pathway discussed here should be viewed as a hypothesis-generating framework rather than a confirmed pathway in FS. Currently, there is no direct evidence from FS tissues or disease models demonstrating that Piezo1-driven YAP/TAZ activation enhances CCL2/CCR2-dependent macrophage recruitment and functional reprogramming. This concept is inferred from mechanistic studies on various fibrotic disorders. Nevertheless, growing clinical and biomechanical data underscore the significant role of mechanical stress in the development of capsular fibrosis in FS. Future research using FS-specific samples and experimental models is required to determine whether this mechanotransduction axis functions in the glenohumeral capsule and to clarify its translational relevance in clinical settings.

## 6 Therapeutic perspectives on modulating macrophage activity in FS

FS is a chronic and progressive fibrotic condition marked by ongoing pain and restricted joint movement, which significantly diminishes the quality of life and hampers functional autonomy. Although some patients experience gradual improvement, many endure prolonged disability and economic burdens. Therefore, symptom relief and shortening of the disease course remain key clinical priorities. Current treatments, including physical therapy, manual manipulation, nonsteroidal anti-inflammatory drugs, intra-articular steroid injections, capsular distension, and arthroscopic synovectomy, are frequently used to reduce pain and moderately improve the glenohumeral function. However, multimodal combinations are often necessary to achieve satisfactory outcomes and involve procedural risks, while lacking strategies that address the underlying fibrotic mechanisms (3, 142, 143). Accumulating evidence implicates macrophages as central regulators of FS pathogenesis and highlights their potential as mechanistic nodes influenced by diverse interventions. Exploring the impact of existing or emerging therapies on macrophage activity may provide a foundation for developing more precise mechanism-oriented treatment strategies in the future. Given the anatomically confined nature of the glenohumeral capsule and spatially localized inflammatory–fibrotic lesions in FS, therapeutic strategies that consider both immunoregulatory mechanisms and delivery context may be particularly relevant.

### 6.1 Therapeutic opportunities in inflammation modulation

Establishment of an inflammatory microenvironment within the capsule is considered a precursor to the onset and progression of FS. Among the various inflammatory factors, TNF-α is widely acknowledged as an essential mediator linking the inflammatory response to tissue damage, and its sustained expression in FS has been confirmed across multiple tissue levels (13, 26). Compared to treatment strategies targeting other inflammatory factors such as IL-1β and IL-6, TNF-α blockers not only benefit from a well-established drug platform and clear immune targets, but also offer a viable pathway for translational exploration in FS, given their successful application in inflammatory bone and joint diseases such as OA and rheumatoid arthritis (RA). This review examines the potential therapeutic implications of anti-inflammatory strategies in FS, with a focus on TNF-α blockade as a representative example.

Adalimumab, a TNF-α inhibitor, has been administered to more than five million patients, demonstrating a favorable safety profile in over 25,000 participants across nine approved indications (144). A randomized clinical trial demonstrated that adalimumab alleviates pain and enhances shoulder function in patients with early-stage FS, where pain is the predominant symptom. These findings indicate that TNF-α blockade may provide a therapeutic window during the early inflammatory phase of FS, although the clinical responses are expected to differ among individuals (144). Subcutaneous administration of adalimumab has shown robust efficacy in systemic inflammatory joint diseases, such as OA and RA (145, 146). However, its effectiveness in FS may be constrained by the unique anatomical and pathological features of the glenohumeral capsule. Supporting this notion, a clinical case report described a patient with RA complicated by severe adhesive capsulitis and diffuse capsular hypertrophy with calcification, in whom long-term subcutaneous adalimumab (40 mg biweekly) effectively controlled peripheral joint inflammation but failed to produce meaningful improvements in shoulder symptoms (147). This discrepancy suggests that systemic adalimumab may have limited penetration or bioavailability within the fibrotic and partially calcified capsule microenvironment. In this context, delivering adalimumab directly into the joint may present a more logical strategy for FS by increasing the local drug concentration at the lesion site while reducing systemic compensation and off-target effects (148).

Therefore, ultrasound-guided intra-articular delivery should be prioritized to improve drug delivery precision and optimize therapeutic outcomes. Mechanistically, the rationale for prioritizing intra-articular delivery is bolstered by converging evidence that inflammatory and fibrotic changes in FS are mainly localized to the capsular synovium and its lining regions, which are directly accessible from the joint space (149–151). This rationale is further supported by evidence from corticosteroid injection studies in FS, which offer a clinically established context to compare how different local administration routes translate into lesion-relevant exposure. In an animal model mimicking the freezing phase, a single intra-articular corticosteroid injection reduced inflammation- and fibrosis-associated protein expression within the capsule, lessened synovial thickening and fibrotic remodeling, and improved shoulder range of motion, reinforcing the concept that direct intra-articular exposure can modulate lesion-proximal pathways in FS (152). In clinical studies, a meta-analysis of randomized controlled trials reported more favorable outcomes with intra-articular corticosteroid injection than with subacromial injection, discussed in relation to achieving higher and more sustained intra-articular drug availability and more direct access to synovial targets (153). Furthermore, a double-blind randomized study suggested that intra-articular injection alone may suffice for many patients, as adding a rotator interval injection did not provide an incremental benefit, possibly because periarticular soft tissue uptake limits effective drug availability at the intended capsular target (154). Overall, these observations underscore that even for the same locally delivered agent, the anatomical route of administration can significantly influence drug exposure at disease-relevant sites. This delivery consideration may help explain why systemically administered biologics with known immunomodulatory capacity, such as adalimumab, show limited clinical benefit in FS and support prioritizing ultrasound-guided intra-articular delivery when evaluating such agents in this anatomically constrained capsular disease.

In this context, the limited clinical response observed with systemic administration does not necessarily imply a deficiency in the immunomodulatory potential of adalimumab. Conversely, mechanistic studies conducted in controlled experimental settings have indicated that adalimumab is capable of modulating macrophage activity. This raises the possibility that its suboptimal performance in FS may be related to insufficient lesion-level exposure, rather than a deficiency in its immunomodulatory potential.

Adalimumab not only exerts anti-inflammatory effects through TNF-α neutralization but also modulates immune activity by reshaping macrophage functional programs. As major producers and responders of TNF-α, macrophages are highly sensitive to changes in cytokine signaling within their microenvironment, and TNF-α blockade can attenuate inflammatory amplification while favoring a shift toward repair-related conditions. Beyond directly neutralizing TNF-α, *in vitro* evidence suggests that adalimumab can actively reprogram the functional state of macrophages. TNF–adalimumab immune complexes can suppress the differentiation of inflammatory M1-like macrophages (CD64^+^ CD80^+^) while preferentially promoting reparative and immunoregulatory M2-like programs. Specifically, adalimumab enhances IL-4–driven M2a polarization, marked by increased expression of CD163, CD206, and CD11b. It also promotes a CD11b^+^ CD206^high^ immunoregulatory macrophage program consistent with M2b features and restores IL-10–driven M2c differentiation (CD11b^+^ CD163^high^), which is otherwise inhibited by TNF. Transcriptomic profiling further revealed a transcriptional signature in macrophages associated with wound healing, marked by controlled matrix degradation and regulated tissue remodeling. This regulatory effect was more pronounced than that of other TNF inhibitors (155). Consistent with these functional effects, clinical studies in RA have shown that adalimumab treatment is associated with a marked reduction in the number of CD68^+^ macrophages in the synovial tissue (156). In parallel, recent evidence indicates that adalimumab can modulate the polarization profile and immune activity of macrophages within the synovial fluid, particularly by attenuating M1-like inflammatory programs. These changes are accompanied by improvements in clinical symptoms while maintaining a favorable tolerance profile throughout the treatment period (145). Collectively, these findings suggest that adalimumab may exert therapeutic effects not only by suppressing inflammatory cytokine signaling but also by redirecting macrophage-mediated matrix remodeling toward a more controlled, repair-oriented program, highlighting a macrophage-centered immunomodulatory mechanism that extends beyond general anti-inflammatory activity.

Nevertheless, the current evidence base remains preliminary and does not permit firm FS-specific conclusions regarding adalimumab. Available studies are limited by small sample sizes, short follow-up periods, and restricted dosing and comparator designs. Most clinical evaluations of FS have prioritized symptomatic endpoints, such as pain relief and range of motion, rather than directly assessing macrophage-centered responses within the capsule. Consequently, it remains difficult to determine whether any clinical benefit reflects lesion-level modulation of macrophage recruitment, functional reprogramming, or matrix-remodeling activity, or represents the downstream effects of broader cytokine suppression. However, these limitations should not be interpreted as negating the translational potential of TNF-α inhibition. Given the demonstrated capacity of adalimumab to reshape macrophage functional programs in controlled settings and its established clinical utility across inflammatory joint diseases, optimizing lesion-level exposure may be a key determinant of FS efficacy. In this regard, ultrasound-guided intra-articular delivery offers a practical strategy to increase local target engagement within the anatomically constrained capsular compartment and may expand therapeutic options for patients, particularly during the early disease phases. Therefore, future FS-oriented studies are warranted to integrate disease-adapted delivery with mechanistic readouts, including macrophage state and matrix-remodeling biomarkers, alongside rigorous assessment of safety and clinical outcomes.

### 6.2 Current evidence and translational opportunities of CCL2/CCR2 axis targeting

Modulation of inflammatory pathways is central to regulating macrophage activity and function in fibrotic diseases. TNF-α blockade is one of the most established anti-inflammatory strategies and exemplifies how macrophage-driven pathology can be modulated through upstream inflammatory control. Based on this concept, targeting macrophage recruitment has emerged as a promising approach. The CCL2/CCR2 signaling axis, which governs macrophage migration and tissue accumulation, has been consistently implicated in fibrotic progression across multiple disease models. Accumulating evidence underscores its value as a therapeutic target with significant translational relevance.

Several candidate drugs targeting CCL2 or CCR2 pathways have advanced to the clinical research phase, offering preliminary insights into the potential for intervention within these pathways. ABN912, a humanized anti-CCL2/MCP-1 monoclonal antibody known for its high affinity and specificity, was tested in a randomized clinical trial involving patients with RA. The trial demonstrated that intravenous infusion of varying doses (0.3, 1, 3, and 10 mg/kg) of ABN912 resulted in a dose-dependent increase in the complex formed by ABN912 and CCL2 in peripheral blood, underscoring the drug’s robust targeting capability *in vivo*. However, the treatment did not lead to statistically significant changes in macrophage counts in the peripheral blood and synovial tissue, and a rebound effect was observed in the high-dose group. Consequently, despite the good tolerance of ABN912 among RA patients, it has not been considered an effective treatment strategy due to the lack of clear clinical improvement or immunohistochemical effects (157).

Subsequently, Vergunst et al. (158) conducted clinical trials on CCR2 blocking antibodies, aiming to achieve optimal therapeutic outcomes by targeting these receptors. They effectively accomplished targeted blockade of CCR2 through intravenous administration of MLN1202, a human anti-CCR2 monoclonal antibody. Although this treatment effectively reduced the level of free CCR2 in peripheral CD14+ monocytes, it did not consistently alleviate inflammation or significantly improve fibrosis markers in synovial tissue. Additionally, the changes in CD22^+^ lymphocytes, CD38^+^ plasma cells, CD163^+^/CD68^+^ macrophages, CD55^+^ synovial fibroblast-like cells, and inflammatory factors (IL-1β, IL-6, TNF-α) expression varied across different dose groups, lacking a dose-dependent response. This pattern suggests that the CCR2 blockade does not effectively suppress the inflammatory cascade in the local synovial environment.

Carlumab, an IgG1κ monoclonal antibody with high specificity for CCL2, effectively reduces circulating levels of free CCL2, and has demonstrated favorable tolerability in clinical applications (159). Pharmacokinetic and pharmacodynamic modeling has shown that carlumab binds quickly to circulating CCL2, forming antibody–chemokine complexes. However, free CCL2 levels only temporarily decrease after infusion, rebounding to baseline or higher within approximately one week, especially with repeated dosing (160). These findings suggest that achieving sustained suppression of circulating CCL2 is challenging under clinically feasible dosing conditions, likely because of the strong compensatory regulation of the CCL2 axis. Reflecting these pharmacokinetic limitations, a phase II randomized trial of carlumab in idiopathic pulmonary fibrosis did not show significant improvement in lung function or clinical outcomes, despite effective target engagement, and a trend toward worsening was noted in the moderate-dose group (5 mg/kg) (161). This result underscores the difficulty in translating systemic CCL2 neutralization into a lasting therapeutic benefit for fibrotic diseases. Beyond clinical outcomes, there are limited mechanistic data on the immune cell–level effects of carlumab. Most studies have focused on changes in circulating or total CCL2 concentrations, with relatively little direct assessment of macrophage recruitment, phenotype, or tissue-level dynamics. The rapid rebound of free CCL2 observed after carlumab administration (160) prompted us to consider the possibility that compensatory chemokine production by resident or infiltrating immune cells may partially offset CCL2 neutralization. However, this interpretation is based on pharmacokinetic observations and has not been directly tested in target tissues. In preclinical settings, carlumab has demonstrated the ability to interfere with CCL2-mediated macrophage recruitment in a nude mouse xenograft model of human endometrial carcinoma, where treatment partially reduced M2-like macrophage infiltration and was associated with restrained tumor growth *in vivo* and in patient-derived organoid systems (162). While these findings support a macrophage-related biological effect of CCL2 neutralization under controlled experimental conditions, caution should be exercised when extrapolating such mechanisms to FS, given the substantial differences between the tumor microenvironment and fibrotic capsular niche. Concurrently, bioinformatics-based analyses have identified CCL2 as a musculoskeletal disease-associated signaling node and highlighted carlumab as a potential candidate for modulating this pathway (163, 164). These computational observations support the conceptual foundation for exploring CCL2-targeted strategies in FS-specific experimental and translational contexts; however, further experimental validation is required.

Although ABN912, MLN1202, and carlumab have shown strong target affinity, their clinical trials failed to achieve the expected therapeutic outcomes, suggesting that single-axis blockade may be insufficient to produce durable benefits owing to compensatory networks. This has prompted a shift toward the simultaneous inhibition of multiple chemotactic pathways. Cenicriviroc, an oral dual CCR2/CCR5 antagonist, was shown in preclinical studies inhibits monocyte and macrophage recruitment and suppresses hepatic stellate cell activation (165). In a subsequent Phase IIb trial, it demonstrated good safety and tolerability over one year, accompanied by reductions in inflammatory markers and improvements in fibrosis, particularly in patients with advanced baseline fibrosis. A two-year extension indicated that these effects could be maintained in some participants (166, 167). However, a large Phase III study did not confirm any histological or clinical benefits, as pharmacodynamic changes such as reductions in peripheral monocyte counts failed to translate into measurable therapeutic outcomes (168).

The CCL2/CCR2 axis is a crucial hub in the recruitment of macrophages, functional remodeling, and the progression of fibrosis. While preclinical research generally supports its potential as a therapeutic target, clinical outcomes indicate that blocking a single target often does not yield consistent efficacy. This suggests that effective treatment might require a higher degree of target engagement or combination with other pathways. In addition to direct antagonism, modulation of upstream inflammatory signals could also indirectly inhibit this axis. As previously mentioned, in the context of TNF-α blockade, such an anti-inflammatory strategy could be advantageous in a combined therapeutic approach. Research has demonstrated that adalimumab treatment for RA can significantly lower serum CCL2 levels, outperforming the standard regimens (169). The mechanism may involve suppression of the MAPK and NF-κB pathways, along with epigenetic modifications in the CCL2 promoter region (170). However, these mechanisms are primarily derived from *in vitro* studies, and their relevance to tissue-level CCL2 inhibition and clinical applications still requires further validation.

Numerous studies have underscored the pivotal role of the CCL2/CCR2 axis in the mechanistic progression of fibrosis; however, its clinical application remains limited. Several factors may contribute to this gap. One consideration is the presence of compensatory responses following CCL2 or CCR2 inhibition, which may manifest as increased chemokine availability or engagement of alternative recruitment pathways, thereby diminishing therapeutic efficacy. In light of this, delivery strategies that ensure prolonged and stable intra-articular target engagement, such as sustained-release formulations, may help mitigate abrupt fluctuations in local CCL2 availability and potentially reduce rebound effects. Supporting this rationale, FS-related studies have demonstrated that intra-articular sustained-release systems, including injectable hydrogels and extended-release formulations, are associated with slower synovial drug release, reduced systemic exposure, and more sustained modulation of local inflammatory and fibrotic activities (171, 172). Additional challenges arise from tissue-specific differences in pathological microenvironments and disease trajectories, which can influence both the timing and responsiveness of chemokine-targeted interventions. Moreover, reliance on systemic or remote administration in many studies may limit effective drug exposure at lesion sites. In this regard, FS, characterized by well-defined anatomical boundaries and spatially confined capsular pathology, provides a favorable context for exploring the localized blockade of the CCL2/CCR2 axis.

In this context, future research should prioritize (1) implementing local blockade strategies during the early stages of the disease to disrupt the positive feedback loop between inflammation and fibrosis; (2) delivering therapeutic agents directly within the glenohumeral capsule or rotator interval to enhance local target engagement while minimizing systemic compensation; (3) exploring combination approaches that integrate local chemokine axis modulation with upstream inflammatory control, such as TNF-α blockade, to achieve more coordinated regulation of macrophage-driven fibrotic processes; and (4) investigating sustained-release delivery strategies, including injectable depots or biomaterial-based carriers, to maintain more stable lesion-level target coverage, potentially mitigating peak–trough fluctuations that may contribute to systemic compensation, while extending local exposure and improving tolerability.

### 6.3 Therapeutic pathways of non-invasive mechanical approaches

Persistent fibrotic remodeling in FS is tightly linked to an aberrant local mechanical environment (25). Mechanical cues not only reflect structural alterations but also actively reshape inflammatory and reparative cellular programs (123, 125, 135). While direct pharmacological targeting of mechanosensing remains constrained by safety and feasibility (173), exogenous modulation of the mechanical microenvironment offers a pragmatic and potentially translatable route to indirectly recalibrate immune–fibrotic responses.

Physical therapy remains the cornerstone of conservative management of FS, primarily alleviating pain and improving mobility through passive stretching to address capsular adhesions (174). In the UK FROST three-arm RCT involving over 500 patients, structured early physiotherapy (combined with a single corticosteroid injection), manipulation under anesthesia, and arthroscopic capsular release produced comparable pain and functional outcomes at 8–12 months, despite differences in early recovery trajectories (174). This supports physiotherapy as a first-line strategy, reserving surgery for cases unresponsive to conservative treatment. The latest guidelines from the British Elbow and Shoulder Society similarly underscore physiotherapy as the foundation for early management. Corticosteroid injections can provide short-term pain relief and improve engagement in therapy; however, its long-term benefit remains uncertain, and repeated injections may increase the risk (175). Therefore, clinical decisions should be individualized, integrating disease stage, joint stiffness, comorbidities, and patient adherence, while reserving escalation to surgery for those who fail structured conservative care.

Although physiotherapy provides proven benefits, its intensity and frequency are often constrained by treatment resources and patient adherence, making it difficult to deliver sufficient mechanical stimulation in the short-term. To address this gap, device-based interventions such as static progressive stretching and high-intensity stretching have been introduced as non-surgical strategies to restore shoulder range of motion (ROM). These interventions enhance the stretch intensity and treatment dosage by providing sustained and controllable external traction, thereby facilitating capsular elongation and tissue release (176). An early small randomized trial showed that both continuous passive stretching and conventional physiotherapy significantly improved ROM after 4 weeks, with similar gains at 12 weeks, whereas passive stretching achieved greater reductions in resting, movement, and nocturnal pain (177). Subsequent studies have demonstrated that combining mechanical stretching with physiotherapy provided superior short-term and sustained long-term improvements in ROM and pain (178). Real-world data from 1,871 patients further supported these findings, showing significant ROM gains across all planes, particularly in those with the lowest baseline ROM, often reducing the requirement for surgery (176).

A recent randomized trial directly compared high-intensity stretching, combination therapy, and physiotherapy alone. ROM recovery exceeded 95% in the stretching-only group and 92% in the combination group, versus 82% in the physiotherapy group. Approximately 30% of physiotherapy-only patients required corticosteroid injections for inadequate pain control, whereas none of the stretching-only group did (179). These findings suggest that when applied with good adherence and standardized protocols, mechanical stretching can improve ROM, reduce pain, and decrease adjunctive drug use. In these clinical studies, standard protocols are increasingly aligned with specific treatment intensities and durations. For instance, high-intensity stretching is commonly recommended for approximately 60 min daily, administered through repeated stretch–rest cycles. In contrast, static progressive stretching protocols generally begin with 30-minute sessions once a day, gradually increasing the frequency over several weeks (178, 179). Similarly, continuous passive motion has been implemented for one hour per day over four weeks in controlled environments (177). Preclinical evidence supports this biological plausibility, showing that stretching improves matrix mechanics and suppresses fibrotic signaling (180). Consistent with this concept, clinical studies have reported that patients undergoing intensified stretching exhibit altered circulating markers of matrix remodeling, including reduced levels of MMP-1 and MMP-2 and increased levels of TIMP-1, TIMP-2, and TGF-β1, accompanied by functional improvement of the shoulder (181). Although these observations do not establish a direct immune-mechanical causal pathway, they suggest that mechanical intervention can influence fibrosis-related molecular programs *in vivo*.

Taken together, evidence indicates that mechanical stretching, whether applied as an adjunct to conservative therapy or as a stand-alone intervention, can significantly improve ROM and shoulder function in the early stages of FS, with effects that are sustained over time. Compared with conventional physiotherapy, it shows greater potential to reduce surgical demand while enhancing safety and accessibility. These clinical benefits are likely to reflect several interconnected mechanisms. First, self-controlled stretching enables patients to adjust intensity and rhythm within individual tolerance, reducing protective muscle responses and fear avoidance, which in turn improves end-range reach and engagement. Second, device-based stretching facilitates high-frequency home use, increasing adherence and cumulative treatment dosage, thereby compensating for the limited intensity of conventional physiotherapy. Finally, progressive traction relieves capsular collagen tension, normalizes abnormal mechanical stress, and improves matrix compliance, potentially supporting microenvironmental homeostasis and attenuating fibrotic remodeling.

Nonetheless, the majority of existing studies are randomized controlled trials, which are vulnerable to subjective influences, such as patient expectation effects and evaluator bias. Future research should emphasize multicenter, large-scale prospective trials. Mechanistic clinical studies that incorporate imaging, molecular, and functional assessments of the glenohumeral joint are necessary to elucidate how exogenous mechanical stimulation influences stress transmission pathways within the capsule, thereby contributing to both the pathogenesis and resolution of FS. Future studies integrating controlled mechanical loading with immune and fibrotic readouts are essential to determine whether these matrix-level and molecular changes are accompanied by macrophage reprogramming within the capsular microenvironment.

## 7 Conclusion and prospect

FS is a musculoskeletal disorder marked by the progressive development of chronic inflammation and fibrosis, ultimately resulting in contraction and adhesion of the glenohumeral capsule. In the initial stages, aseptic inflammation within the capsule prompts the sustained release of cytokines and chemokines, which subsequently induces fibroblast activation and abnormal ECM accumulation. As the condition progresses, inflammatory signals and fibrosis remodeling mutually reinforce each other and gradually establish a pathological circuit that is challenging to self-limit. Throughout this process, macrophages play a pivotal role as central hubs linking the amplification of inflammation and progression of fibrosis, exhibiting clear situational dependence and stage-specific responses. Their recruitment, polarization, and functional status are synergistically regulated by various microenvironmental factors, including inflammatory mediators, chemotactic signals, and mechanical stimuli, and they display distinct temporal patterns. Recognizing this dynamism can facilitate the identification of intervention windows at specific disease stages.

While previous research has underscored the critical pathological role of macrophages in FS, the mechanisms driving the phenotypic switching of these communities and their functional patterns across different pathological environments require further exploration. A key unresolved question is not only which macrophage programs are involved, but also where these programs are located within the capsular lesion and how their spatial organization correlates with the transition from inflammation to fibrosis. Spatially resolved multiomics marks a significant methodological advancement that enables the coordinated assessment of multiple molecular layers, such as transcripts, proteins, and tissue architecture, within intact tissue sections. By maintaining spatial context, this approach allows for the delineation of lesion microdomains and direct inference of immune–stromal neighborhood relationships *in situ*. When paired with artificial intelligence (AI)-assisted analytical frameworks, these high-dimensional spatial datasets can be systematically integrated through automated spatial clustering, domain identification, and cell–cell interaction inference, thereby enhancing their scalability, reproducibility, and interpretability across samples (182). In the context of FS, spatial multiomics could potentially elucidate whether distinct macrophage subsets preferentially localize to specific capsular regions, such as the synovial lining, subsynovial layer, or areas of significant ECM accumulation and increased stiffness, and whether these cells are spatially adjacent to fibroblasts or other immune populations. Furthermore, comparisons across disease stages may provide insights into whether inflammatory and fibrotic programs tend to occupy separate capsular regions or emerge sequentially within the same microenvironment, and whether the dominant pathological focus shifts spatially as FS progresses. Through AI-enabled integration, such spatially anchored information may support more refined, stage-relevant mechanistic models and help guide the prioritization of locally targeted and stratified intervention strategies for FS.

Despite these advances, the current understanding of macrophage mechanisms in the progression from inflammation to fibrosis remains incomplete, resulting in interventions that primarily focus on blocking inflammation or inhibiting recruitment, without precise regulatory strategies for their functional status and dynamic evolution. Existing evidence indicates that relying solely on pharmacological approaches to block inflammation or inhibit macrophage recruitment is insufficient for reversing existing tissue fibrosis. Future research should focus on the precise reprogramming and local regulation of pathogenic macrophage subpopulations at various stages of disease. This approach aims to minimize systemic adverse reactions while enhancing therapeutic specificity. Achieving this will likely require engineered platforms capable of spatially confined and temporally controlled modulation within the capsular microenvironment. Recent advancements in bioengineering indicate that controllable, locally retained delivery systems may provide a practical means of achieving this modulation. Microsphere-based carriers offer a promising approach for translating localized and sustained drug delivery concepts into FS treatment. Biodegradable polymer microspheres can achieve high drug-loading efficiency and programmable release kinetics, allowing therapeutic agents to remain within the target tissue for extended periods while minimizing systemic exposure (183). These features are particularly relevant for FS, where pathological inflammation and fibrosis are confined to the glenohumeral capsule, and short-lived intra-articular exposure may not be sufficient for durable immunomodulatory effects. Notably, microelectromechanical system–based bioprinting has been proposed as a scalable fabrication method to enhance microsphere uniformity and batch-to-batch consistency, potentially leading to more predictable release profiles and improving the translational feasibility of localized sustained-release strategies in capsular diseases (183). Simultaneously, advances in three-dimensional (3D) printing for tissue engineering, particularly in bone regeneration, illustrate a broader principle in which material composition and structural design can be deliberately programmed to jointly modulate mechanical cues and biologically active signals. Studies using polylactic acid/nano-hydroxyapatite composite scaffolds further modified with bioactive ions, such as magnesium or lithium, have shown that precisely engineered material properties can be coupled with pathway-level activation of osteogenic and angiogenic programs, supporting the concept of 3D bioprinting as a platform for microenvironmental regulation beyond structural fabrication (184–186). Although FS is characterized by fibrotic remodeling rather than tissue loss, the glenohumeral capsule remains a mechanically sensitive soft-tissue compartment that requires dynamic compliance under complex loading. Therefore, these engineering concepts may inform FS-oriented designs of minimally invasive, soft-tissue-compatible biomaterials that combine controlled drug loading and sustained local release with modulation of lesion mechanics, potentially creating conditions that are more permissive for stage-appropriate macrophage remodeling. In future FS-oriented investigations, incorporating macrophage-centered readouts alongside conventional clinical endpoints may provide a more informative framework for assessing the biological impact of such platforms.

It is crucial to emphasize that local biomechanical steady-state disruption and increased capsular stiffness significantly contribute to the progression of FS. However, effective targeted strategies that directly address these biomechanical abnormalities are lacking. Current FS management primarily relies on invasive procedures, which are associated with procedural risks, limited durability of benefits, and substantial economic burden. Therefore, reducing systemic exposure and procedural risks while developing more adaptable and stage-sensitive treatment strategies remains a key goal for improving FS care. Non-invasive interventions, particularly device-based mechanical stretching, offer advantages in terms of repeatability, safety, and patient adherence, making them attractive candidates for broader clinical adoption in FS. Beyond their mechanical effects, such approaches may indirectly influence the local immune microenvironment by improving tissue compliance and restoring the capsular mechanical balance. Recent advances in digital health research have further highlighted how the integration of flexible sensing technologies with AI can enhance rehabilitation by enabling continuous physiological monitoring, data-driven adjustments, and scalable remote management. Flexible sensors can capture high-sensitivity biomechanical and electrophysiological signals over time, whereas AI-based analytical frameworks are well-suited for interpreting the resulting complex and high-dimensional datasets, thereby supporting adaptive control and individualized rehabilitation strategies (187). Against this backdrop, the application of AI-assisted flexible sensing may hold particular promise for refining non-invasive mechanical interventions in FS. When incorporated into device-based stretching systems, such platforms could potentially enable real-time detection of insufficient or excessive mechanical loading, support individualized adjustment of stretching intensity and frequency, and characterize patient-specific response patterns across the treatment and recovery phases. Coupled with remote data integration and cloud-based management, these technologies may also facilitate home-based rehabilitation and longitudinal monitoring, with potential benefits for the accessibility, adherence, and personalization of FS care. Nevertheless, although non-invasive mechanical stretching is already applied in FS management, the biological mechanisms through which mechanical interventions influence macrophage behavior and fibrotic remodeling within the capsular microenvironment remain insufficiently understood. Whether emerging sensor-assisted and AI-supported rehabilitation systems can meaningfully enhance these mechanobiological effects remains an open question, highlighting the need for future studies that integrate mechanical, immunological, and functional readouts in FS.

Overall, staged research integrating immunology and mechanobiology, along with emerging bioengineering and digital health approaches, has the potential to yield more actionable and precise intervention strategies for FS, providing a conceptual framework that links fundamental mechanisms with clinically translatable solutions. .

## Glossary

FS: frozen shoulder
M1: classically activated macrophage
M2: alternatively activated macrophage
MHC-II: major histocompatibility complex class II
CCL2: C-C motif chemokine ligand 2
CCR2: C-C chemokine receptor type 2
CX3CR1: CX3C chemokine receptor 1
IL: interleukin
Th17: T helper 17 cell
IL-17RA: interleukin-17 receptor A
TNF-α: tumor necrosis factor alpha
TGF-β: transforming growth factor beta
TGF-βR1: transforming growth factor beta receptor 1
Smad: mothers against decapentaplegic homolog
ECM: extracellular matrix
α-SMA: alpha-smooth muscle actin
COL1: collagen type I
MMP: matrix metalloproteinase
TIMP: tissue inhibitor of metalloproteinases
PDPN: podoplanin
PDGF: platelet-derived growth factor
VEGF: vascular endothelial growth factor
FGF: fibroblast growth factor
VCAM: vascular cell adhesion molecule
POSTN: periostin
PI3K: phosphatidylinositol 3-kinase
Akt: protein kinase B
p38 MAPK: p38 mitogen-activated protein kinase
NF-κB: nuclear factor kappa-B
COX: cyclooxygenase
S100A8/9: S100 calcium-binding protein A8/A9
ROS: reactive oxygen species
HMGB1: high-mobility group box 1
MERTK: MER tyrosine kinase
PGE2: prostaglandin E2
RAGE: receptor for advanced glycation end products
Piezo: piezoelectric ion channel
YAP: yes-associated protein
TAZ: transcriptional co-activator with PDZ-binding motif
TEAD: TEA domain transcription factor
ROM: range of motion
OA: osteoarthritis
RA: rheumatoid arthritis. TRPV4, transient Receptor Potential Vanilloid 4
PKD2: polycystin-2
Arg-1: Arginase-1
MERTK: MER proto-oncogene, tyrosine kinase
AI: artificial intelligence
3D: three-dimensional.
